# The impact of comorbid severe mental illness and common chronic physical health conditions on hospitalisation: A systematic review and meta-analysis

**DOI:** 10.1371/journal.pone.0272498

**Published:** 2022-08-18

**Authors:** Naomi Launders, Kate Dotsikas, Louise Marston, Gabriele Price, David P. J. Osborn, Joseph F. Hayes

**Affiliations:** 1 Division of Psychiatry, UCL, London, United Kingdom; 2 Department of Primary Care and Population Health, UCL, London, United Kingdom; 3 Health Improvement Directorate, Public Health England, London, United Kingdom; 4 Camden and Islington NHS Foundation Trust, St Pancras Hospital, London, United Kingdom; Universita degli Studi di Milano-Bicocca, ITALY

## Abstract

**Background:**

People with severe mental illness (SMI) are at higher risk of physical health conditions compared to the general population, however, the impact of specific underlying health conditions on the use of secondary care by people with SMI is unknown. We investigated hospital use in people managed in the community with SMI and five common physical long-term conditions: cardiovascular diseases, COPD, cancers, diabetes and liver disease.

**Methods:**

We performed a systematic review and meta-analysis (Prospero: CRD42020176251) using terms for SMI, physical health conditions and hospitalisation. We included observational studies in adults under the age of 75 with a diagnosis of SMI who were managed in the community and had one of the physical conditions of interest. The primary outcomes were hospital use for all causes, physical health causes and related to the physical condition under study. We performed random-effects meta-analyses, stratified by physical condition.

**Results:**

We identified 5,129 studies, of which 50 were included: focusing on diabetes (n = 21), cardiovascular disease (n = 19), COPD (n = 4), cancer (n = 3), liver disease (n = 1), and multiple physical health conditions (n = 2). The pooled odds ratio (pOR) of any hospital use in patients with diabetes and SMI was 1.28 (95%CI:1.15–1.44) compared to patients with diabetes alone and pooled hazard ratio was 1.19 (95%CI:1.08–1.31). The risk of 30-day readmissions was raised in patients with SMI and diabetes (pOR: 1.18, 95%CI:1.08–1.29), SMI and cardiovascular disease (pOR: 1.27, 95%CI:1.06–1.53) and SMI and COPD (pOR:1.18, 95%CI: 1.14–1.22) compared to patients with those conditions but no SMI.

**Conclusion:**

People with SMI and five physical conditions are at higher risk of hospitalisation compared to people with that physical condition alone. Further research is warranted into the combined effects of SMI and physical conditions on longer-term hospital use to better target interventions aimed at reducing inappropriate hospital use and improving disease management and outcomes.

## Introduction

People with severe mental illness (SMI) have more physical health comorbidities [1–5] and poorer prognoses from those comorbidities [6] than the general population. Physical health comorbidities can lead to reduced quality of life [7], worsening mental health [8], and drives excess mortality in people with SMI [9, 10].

Previous systematic reviews have found that people with SMI are at a higher risk of 30-day readmissions compared to those without SMI [11, 12], and that those with SMI and physical health comorbidities are at higher risk of psychiatric admissions compared to those with SMI alone [13].

Studies based on hospital records alone have found that people with SMI use hospitals for physical health more frequently than people without SMI for emergency admissions [14], preventable admissions [15] and all-cause admissions [16]. However, without accounting for underlying physical comorbidities, whether this represents inappropriate use of services is unclear. A recent meta-analysis by Ronaldson et al. [17] found that in studies controlling for physical health comorbidities there were more hospitalisations, ED visits and longer length of stays in people with SMI compared to those without SMI, suggesting the higher service use is not explained by higher prevalence of physical health conditions alone.

The relationship between physical and mental health and the effect on service utilisation is likely complex, dependent on a range of patient and provider factors. Known drivers of hospital utilisation in the general population, such as poor medication adherence, polypharmacy [18] or inappropriate prescribing [19], continuity of care, and patient satisfaction [20–22] may influence hospital utilisation differently depending on the number and type of underlying mental and physical health conditions in a population.

In order to understand the effect of having both a diagnosis of SMI and of physical health conditions on hospital utilisation, we undertook a systematic review and meta-analysis of observational hospital utilisation studies, comparing people with SMI and one of five common physical long-term conditions (LTCs), compared to those with either SMI or LTCs alone. These diseases (cardiovascular diseases, chronic obstructive pulmonary disease (COPD), cancers, diabetes and liver disease) were chosen because of their high burden of disease globally and/or their impact on those with SMI.

## Methods

### Search strategy

We searched the following sources on 24 March 2020 for publications or grey literature within the remit of the study without date restrictions: PubMED, EmBase, Web of Science, PsychInfo, PsychExtra, Health Management Information Centre. Searches for new publications were performed on 17 December 2020 and 17 March 2022. Searches included terms for severe mental illness, physical health conditions and hospitalisation (S1 Appendix). We performed forward and backward citation searching of relevant studies, reviews and editorials. Where conference abstracts were identified searches for related articles were performed. Conference abstracts were excluded from the final analysis, though those with available data were included in a sensitivity analysis. The study protocol was registered with Prospero: CRD42020176251.

### Outcomes

The primary outcomes were planned or unplanned hospital admissions, for either all-causes, all physical health causes, causes specific to the physical LTC under study, or ambulatory care sensitive conditions (ACSC), a list of conditions for which emergency admission is thought to be avoidable [23]. Secondary outcomes were readmissions and attendance at EDs or other acute outpatient care for these causes.

### Inclusion and exclusion criteria

We included observational studies of adults under the age of 75, managed in the community, and diagnosed with SMI and at least one of the physical LTCs of interest (cardiovascular diseases, COPD, cancers, diabetes and liver disease). We defined SMI as patients with a diagnosis of either schizophrenia, bipolar disorder or other non-organic long-term psychotic disorders, in line with the Quality Outcomes Framework used by the NHS in England [24]. We therefore excluded studies that included major depression in their definition of SMI, without stratifying results by mental health condition.

We excluded studies without comparator populations, interventional studies, and reviews. We also excluded studies focused solely on children and young people (under 18) or the elderly (over 75 years), or in populations not managed in the community. We excluded studies focused on planned outpatient care, preventative services such as cancer screening where the setting of service provision was unclear and context specific, and studies focused on admissions for specific procedures. Finally, we excluded studies where the outcome was hospitalisation for a specific physical health condition other than the physical LTC of interest.

### Data screening and extraction

We collated the results of the literature search using EndNote X9 (Clarivate Analytics, PA, USA) and removed duplicates. The first researcher (NL) screened titles and abstracts against inclusion and exclusion criteria in Microsoft Access, and records obtained in March 2020 (70%) were screened by the second researcher (KD). We resolved disagreements through discussion and calculated the Kappa statistic for inter-rater agreement. We acquired full text articles for all studies identified for inclusion which were screened by the first researcher and a 20% sample was screened by the second researcher. We extracted data from included studies using a standardised form, which was piloted on a sub-set of articles prior to finalisation. This form included variables describing the study focus (exposure, outcome, study population, location); design (methodology, effect measure and size, matching or adjusting variables, follow up time, study period), and publication (publication year).

### Statistical analysis

We analysed the data both as a narrative synthesis, and a meta-analysis stratified by physical LTCs. Studies providing adjusted odds ratios (OR) or hazard ratios (HR) were included in the meta-analyses. Pooled OR and HR were calculated on aggregate data and the relationship between SMI and physical health and secondary care utilisation quantified using a random effects meta-analysis, performed in R [25] and R Studio [26]. In-study bias was be assessed using Newcastle-Ottawa scale (NOS) assessment for observational studies. We assessed publication bias by visual scrutiny of funnel plots of effect size against standard error, and where more than ten studies were considered, using an Egger’s test. Study heterogeneity was measured using the I^2^ statistic [27]. We undertook subgroup analysis to account for SMI diagnosis group and outcome measures. Where differences were found between groups in subgroup analysis, meta-regression was performed to determine the effect of controlling for these groups on heterogeneity. We performed a sensitivity analysis using three-level hierarchical meta-analysis. This method allows for the inclusion of multiple results from single studies, accounting for variance between participants and between studies as in random effects meta-analysis, but also the variance between multiple effect sizes within a study [28].

## Results

**Fig 1:**
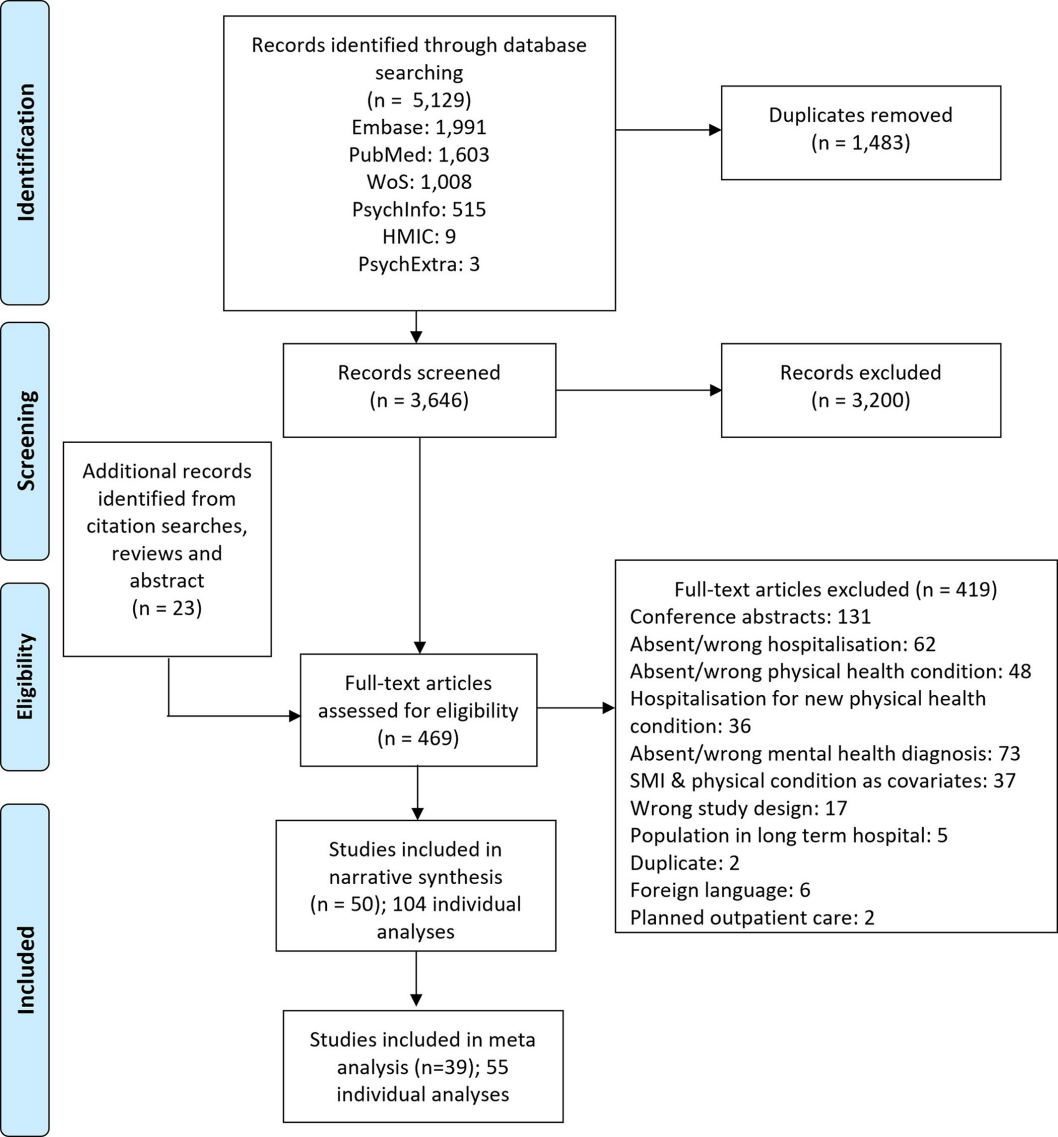
PRISMA flow chart. WoS: Web of Science; HMIC: Health management information consortium.

We identified 5,129 records, of which 3,646 remained after deduplication (Fig 1). Inter-rater agreement of title and abstract screening was 91.4%, with a Kappa statistic of 0.57. Following screening, 50 studies [29–78] were included in the narrative synthesis, published between 2006 and 2022 (Table 1).

**Table 1:** Study description.

Authors	Pub year	Study design	Exposure	Outcome	Population	Notes	Study period	Follow up	Pop size	Unit of measure	Country	Area	Age	Matched
	**Studies of diabetes and SMI**													
Egglefield et al. [29]	2020	Cross sectional	Antipsychotic adherence	Preventable diabetes admissions	Medicaid registered patients with diabetes	Unadjusted data provided for patients with schizophrenia	2012	1 year	191,521	Person	US	One region	18–64	No
Helmer et al. [30]	2020	Cohort	SMI and other MH conditions	Any, acute and chronic ACSC admissions	Veterans Affairs registered patients with diabetes		2010	1 year	151,614	Person	US	National	>66	No
Stockbridge et al. [31]	2019	Cross sectional	Schizophrenia, bipolar disorder, and other MH conditions	Diabetes admissions	Insured patients with diabetes		2011–2013	3 years	229,039	Person	US	National	20–64	No
Tsai et al. [32]	2019	Cohort	Bipolar disorder	Hyperglycaemia admissions	Patients with diabetes		1999–2013	Up to 11 years	30,477	Person	Taiwan	National	Adults	Yes
Goueslard et al. [33]	2018	Cohort	Schizophrenia	Acute diabetes complications long-term readmissions	Patients with type 1 diabetes		2009–2012	3 years	45,655	Person	France	National	15–35	No
Edwards et al. [34]	2014	Cohort	Home-Based Primary Care	ACSC admissions	Veterans Affairs registered patients with diabetes	Psychosis is a covariate	2006–2010	Up to 5 years	56,608	Person	US	National	>67	No
Druss et al. [35]	2012	Cross sectional	Schizophrenia, bipolar disorder, and other MH conditions	ACSC admissions	Medicaid registered patients with diabetes		2003–2004	2 years	657,628	Person	US	National	< = 65	No
Leung et al. [36]	2011	Cohort	Schizophrenia, bipolar disorder, and other MH conditions	Diabetes admissions	Medicaid or medicare registered patients with type 2 diabetes		2005	1 year	106,174	Person	US	One region	>18	No
Mai et al. [37]	2011	Cohort	Schizophrenia, affective psychosis, other psychoses, and other MH conditions	Diabetes admissions	Patients with diabetes		1990–2006	Up to 15.5 years	43,671	Person	Australia	One region	>18	Yes
Cramer et al. [38]	2010	Cross sectional	Risk factors and comorbidities	More than one all-cause long-term readmission	Medicaid registered patients with diabetes	Psychosis is one of many risk factors considered	2005	1 year	695	Person	US	National	Adults	No
Yan et al. [39]	2019	Cohort	Risk factors and comorbidities	All-cause admissions	Patients with antipsychotic-treated schizophrenia, bipolar 1 disorder or major depressive disorder	Type 2 diabetes is one of many risk factors considered	2013–2016	1 year	38,195	Person	US	Multiple regions	>18	No
Chen et al. [40]	2012	Cohort	Outpatient quality of care	All-cause 30-day readmissions	Commercially insured patients with diabetes	Psychosis is a covariate	2010	30 days	30,139	Person	US	National	>19	No
Guerrero Fernandez de Alba et al. [41]	2020	Cohort	Schizophrenia, and other MH conditions	All-cause and diabetes admissions and ED attendances	Patients with type 2 diabetes		2012	1 year	63,365	Person	Spain	One region	>18	No
Chwastiak et al. [42]	2014	Cohort	SMI	All cause 30-day and long-term readmissions	Patients with diabetes		2010–2011	30 days / up to 2 years	82,060	Person	US	One region	>18	No
Becker et al. [43]	2011	Cohort	Schizophrenia	Hyperglycaemia or hypoglycaemia admissions or ED attendances	Patients with diabetes		1996–2006	1–10 years	5,033	Person	Canada	One region	18–50	Yes
Krein et al. [44]	2006	Cross sectional	SMI	All-cause admissions	Veterans Affairs registered patients with diabetes		1997–1998	1 year	36,546	Person	US	National	Mean 58	Yes
Kurdyak et al. [45]	2017	Cohort	Schizophrenia	Diabetes and all-cause admissions and ED attendances	Patients with diabetes		2011–2013	2 years	1,131,375	Person	Canada	One region	19–105	No
Shim et al. [46]	2014	Cohort	Schizophrenia or diabetes	Diabetes and all-cause ED attendances	Medicaid registered patients with diabetes and/or schizophrenia		2006–2007	2 years	432,112	Person	US	Multiple regions	18–64	No
Sullivan et al. [47]	2006	Cross sectional	Bipolar disorder, and other MH conditions	Admissions in those attending ED for diabetes	Patients with diabetes		1994–1998	4.5 years	4,275	Admissions	US	Single site	>18	No
Wang et al. [78]	2021	Cohort	SMI	All cause admissions	Patients with diabetes		2000–2016	6.4 years	6,383	Person	UK	England	>18	Yes
Huang et al. [73]	2021	Cohort	Schizophrenia	All cause admissions	Patients with diabetes		2002–2013	11	10,604	Person	Taiwan	National	Not given	Yes
	**Studies of cardiovascular disease and SMI**													
Attar et al. [48]	2020	Cohort	Schizophrenia	Major adverse cardiac event long-term readmissions	Patients with acute myocardial infarction		2000–2018	5 years	286,333	Person	Sweden	National	>18	No
Chamberlain et al. [49]	2017	Cohort	Multimorbidity	All-cause long-term readmissions	Patients with atrial fibrillation	Schizophrenia is one of many risk factors considered	2000–2014	Up to 14 years	2,860	Person	US	One region	>18	No
Sayers et al. [50]	2007	Cross sectional	Psychosis, bipolar disorders, and other MH conditions	All-cause long-term readmissions	Medicare registered patient with congestive heart failure		1999	1 year	21,429	Person	US	National	65+	No
Shah et al. [51]	2018	Cross sectional	Risk factors and comorbidities	All-cause 30-day readmissions	Patients with non-acute myocardial infarction cardiogenic shock	Psychosis is one of many risk factors considered	2013–2014	30 days	24,665	Person	US	Multiple regions	>16	No
Pham et al. [52]	2019	Cross sectional	Risk factors and comorbidities	All-cause and heart failure 7- and 30-day unplanned readmissions	Medicare registered patient with heart failure	Psychosis is one of many risk factors considered	2014	30 days	234,298	Admissions	US	Multiple regions	>65	No
Chamberlain et al. [53]	2018	Cross sectional	Risk factors and comorbidities	Heart failure 30-day readmissions	Patients with heart failure	Psychosis is one of many risk factors considered	2006–2011	30 days	1,007,807	Person	US	Multiple regions	Not given	No
Shah et al. [54]	2018	Cross sectional	Risk factors and comorbidities	All cause 31-day readmissions	Patients with Takotsubo cardiomyopathy	Psychosis is one of many risk factors considered	2013–2014	31 days	5,997	Person	US	Multiple regions	>18	No
Shah et al. [55]	2018	Cross sectional	Risk factors and comorbidities	All-cause 30-day readmissions	Patients with acute myocardial infarction and cardiogenic shock	Psychosis is one of many risk factors considered	2013–2014	30 days	26,016	Person	US	Multiple regions	>16	No
Jorgensen et al. [56]	2017	Cohort	Schizophrenia	All-cause 28-day readmissions	Patients with heart failure		2004–2013	28 days	36,718	Person	Denmark	National	>18	No
Ahmedani et al. [57]	2015	Cohort	Bipolar disorders, schizophrenia-spectrum disorders, other psychoses, and other MH conditions	All-cause 30-day readmissions	Patients with heart failure or myocardial infarction		2009–2011	30 days	123,921	Admissions	US	Multiple regions	>18	No
Coffey et al. [58]	2012	Cross sectional	Risk factors and comorbidities	Congestive heart failure 30-day readmissions	Patients with congestive heart failure	Psychosis is one of many risk factors considered	2006	30 days		Admissions	US	Multiple regions	>18	No
Lu et al. [59]	2017	Cohort	Schizophrenia, bipolar mood disorder, and other MH conditions	Heart failure 30-day and long-term readmissions	African American patients with heart failure		2010–2013	30 days / ave 3.2 years	611	Person	US	Single site	>20	No
Kallio et al. [69]	2022	Cohort	Schizophrenia	Stroke and myocardial infarction long term readmissions	Patients with coronary artery disease who underwent coronary artery bypass grafting surgery		2004–2018	Up to 10 years	29,220	Person	Finland	Multiple sites	Not given	Yes
Fleetwood et al. [71]	2021	Cohort	Schizophrenia and bipolar disorder	Stroke and myocardial infarction long term readmissions	Patients hospitalised with myocardial infarction		1999–2018	Up to 20 years	184,134	Person	UK	Scotland	>18	No
Ghani et al. [72]	2021	Cohort	SMI	All-cause 30-day emergency readmissions	Patients who underwent vascular surgery		2007–2018	30 days	8,973	Person	UK	One region	>18	No
Fleetwood et al. [70]	2021	Cohort	Schizophrenia and bipolar disorder	Stroke and myocardial infarction long term readmissions	Patients hospitalised with stroke		1991–2018	Up to 28 years	169,923	Person	UK	Scotland	>18	No
Paredes et al. [75]	2020	Cohort	SMI	All-cause 30-day readmissions	Medicare registered patients who underwent coronary artery bypass grafting surgery		2013–2017	30 days	118,837	Person	US	National	>65	No
Sreenivasan et al. [76]	2022	Cohort	Bipolar disorder and schizophrenia or other psychotic illnesses	All-cause 30-day readmissions	Patients hospitalised with myocardial infarction		2016–2017	30 days	904,575	Person	US	National	>18	No
Andres et al. [77]	2012	Cross sectional	Schizophrenia	Long-term readmission for myocardial infarction	Patients hospitalised with myocardial infarction		2000–2007	8 years	19,016	Person	Spain	One region	>15	No
	**Studies of COPD and SMI**													
Buhr et al. [60]	2019	Cross sectional	Charlson and Elixhauser indicies	All-cause 30-day readmissions	Patients with COPD	Psychosis included in the Elixhauser index	2010–2016	30 days	1,622,983	Admissions	US	National	>40	No
Jorgensen et al. [61]	2018	Cohort	Schizophrenia	All-cause 30-day readmissions	Patients with COPD		2008–2013	30 days	211,868	Person	Denmark	National	>30	No
Lau et al. [62]	2017	Cross sectional	Risk factors and comorbidities	COPD 30-day readmissions	Patients with COPD	Psychosis is one of many risk factors considered	2006–2011	30 days	597,502	Person	US	Multiple regions	>40	No
Singh et al. [63]	2016	Cohort	Psychosis, and other MH conditions	All-cause 30-day readmissions	Medicare registered patients with COPD		2001–2011	30 days	135,498	Admissions	US	National	>66	No
	**Studies of cancer, liver disease or multiple diseases and SMI**													
Basta et al. [64]	2016	Cohort	Risk factors and comorbidities	Complicated lymphedema long-term readmissions	Women who had undergone breast cancer related mastectomy /lumpectomy	Psychosis is one of many risk factors considered	2007–2012	2 years	56,075	Person	US	Multiple regions	>18	No
Kashyap et al. [68]	2021	Cohort	Bipolar and psychoses	All-cause 30-day ED attendance	Medicare registered patients with gastrointestinal malignancies in the last 30 days of life		2004–2014	30 days	110,325	Person	US	National	>66	No
Ratcliff et al. [74]	2021	Cohort	Bipolar disorder and psychoses	All cause 90-day readmissions	Veterans Affairs registered patients who underwent surgery for colorectal cancer		Not given	90 days	50,611	Person	US	National	Not given	No
Huckans et al. [65]	2010	Cohort	Schizophrenia	All-cause readmissions during anti-viral therapy	Veterans Affairs registered patients with hepatitis C		1998–2006	During antiviral therapy	60	Person	US	Multiple regions	Mean 50	Yes
Davydow et al. [66]	2016	Cohort	SMI	ACSC admissions	General population	Table 1 provides unadjusted effect for patients with underlying cardiovascular disease, diabetes, liver disease and cancer	1999–2013	14 years	5,945,540	Person	Denmark	National	>18	No
Guo et al. [67]	2008	Cohort	Risk factors and comorbidities	All-cause admissions and ED attendances	Commercially insured patients with bipolar disorder	Diabetes, COPD and heart disease are some of many risk factors considered	1998–2002	Up to 5 years	67,862	Person	US	Multiple regions	Mean 37.1	No

### Study characteristics

Most studies were conducted in the United States (US) (n = 33; Table 1). Forty-four studies quantified the risk of admissions, readmissions or ED visits in a patient population (median population size: 53,343; interquartile range (IQR): 23,856–185,981); while in five studies the focus was the number of index admissions which resulted in a readmission (median admissions: 184,898, IQR: 132,604–581,469), and one investigated the admission ratio of 4,275 ED visits. The majority of studies (n = 38) included adults with an age range of 20 to 65 or wider, while seven focused on those over the age of 65. The remaining studies excluded patients under the age of 30 or 40 (n = 3), those over the age of 50 (n = 1) or those over 35 (n = 1). The included studies were heterogeneous in population, exposure, outcome, and effect measure and 27 could be stratified into multiple analyses based on these factors (Table 2). Of the 104 unique analyses, 59 investigated inpatient admissions over at least a year, with a median follow up of five years (IQR: 2–14). A further 27 investigated inpatient admissions limited to a 28 to 31 day period following an index admission (termed 30-day readmissions) and 12 investigated ED visits (median follow up: 2 years, IQR: 2–5 years). Two analyses investigated 7-day readmissions, two investigated 90-day readmissions, one combined inpatient admissions and ED visits over a ten year period, and one calculated the odds of admission in those attending an ED (Table 2). ED use was the only acute outpatient care outcome identified, and we did not identify any studies of planned inpatient admissions.

**Table 2:** Description of analyses.

Authors	Year	Baseline condition	Exposure	Utilisation	Utilisation type	NOS score	Adjusted for age and sex	Adjusted for physical comorbidities	Adjusted for prior utilisation	Effect measure	Effect size	95%CI/p-value	Included in meta-analysis
**The effect of diabetes on hospital utilisation in patients with SMI**													
Yan et al. [39]	2019	Schizophrenia	Diabetes T2	Inpatient	All cause	9	Yes	Yes	Yes	aOR	1.19	1.05–1.36	NA
Shim et al. [46]	2014	Schizophrenia	Diabetes T1/T2	ED	All cause	4	No	No	No	OR	1.46	1.41–1.51	NA
Yan et al. [39]	2019	Bipolar	Diabetes T2	Inpatient	All cause	9	Yes	Yes	Yes	aOR	1.23	1.13–1.34	NA
Guo et al. [67]	2008	Bipolar	Diabetes	Inpatient	All cause	6	Yes	Yes	No	aRR	1.44	1.36–1.52	NA
Guo et al. [67]	2008	Bipolar	Diabetes	ED	All cause	6	Yes	Yes	No	aRR	1.17	1.08–1.25	NA
**The effect of cardiovascular disease on hospital utilisation in patients with SMI**													
Guo et al. [67]	2008	Bipolar	Ischemic heart disease	Inpatient	All cause	6	Yes	Yes	No	aRR	1.89	1.78–2.02	NA
Guo et al. [67]	2008	Bipolar	Ischemic heart disease	ED	All cause	6	Yes	Yes	No	aRR	1.67	1.53–1.81	NA
**The effect of COPD on hospital utilisation in patients with SMI**													
Guo et al. [67]	2008	Bipolar	COPD	Inpatient	All cause	6	Yes	Yes	No	aRR	1.94	1.81–2.06	NA
Guo et al. [67]	2008	Bipolar	COPD	ED	All cause	6	Yes	Yes	No	aRR	1.61	1.47–1.76	NA
**The effect of SMI on hospital utilisation in patients with diabetes**													
Stockbridge et al. [31]	2019	Diabetes T1/T2	Bipolar	Inpatient	Diabetes	7	Yes	Yes	No	aOR	0.99	0.78–1.25	Yes
Druss et al. [35]	2012	Diabetes T1/T2	Bipolar	Inpatient	ACSC	7	Yes	Yes	No	aOR	1.03	0.98–1.09	Yes
Leung et al. [36]	2011	Diabetes T2	Bipolar	Inpatient	Diabetes	7	Yes	No	Yes	aOR	1.07	0.91–1.26	Yes
Chen et al. [40]	2012	Diabetes T1/T2	Psychosis	30-day	All cause	8	Yes	Yes	Yes	aOR	1.15	1.03–1.29	Yes
Stockbridge et al. [31]	2019	Diabetes T1/T2	Schizophrenia	Inpatient	Diabetes	7	Yes	Yes	No	aOR	1.61	1.29–2.01	Yes
Goueslard et al. [33]	2018	Diabetes T1	Schizophrenia	Inpatient	Diabetes	6	Yes	Yes	No	aOR	2.21	1.69–2.88	Yes
Druss et al. [35]	2012	Diabetes T1/T2	Schizophrenia	Inpatient	ACSC	7	Yes	Yes	No	aOR	1.26	1.21–1.30	Yes
Leung et al. [36]	2011	Diabetes T2	Schizophrenia	Inpatient	Diabetes	7	Yes	No	Yes	aOR	0.75	0.63–0.89	Yes
Guerrero Fernandez de Alba et al. [41]	2020	Diabetes T2	Schizophrenia	Inpatient	All cause	6	Yes	Yes	No	aOR	1.40	1.18–1.66	Yes
Guerrero Fernandez de Alba et al. [41]	2020	Diabetes T2	Schizophrenia	Inpatient	Diabetes	6	Yes	Yes	No	aOR	1.25	0.55–2.82	Yes
Guerrero Fernandez de Alba et al. [41]	2020	Diabetes T2	Schizophrenia	ED	All cause	6	Yes	Yes	No	aOR	1.28	1.11–1.47	Yes
Kurdyak et al. [45]	2017	Diabetes T1/T2	Schizophrenia	ED	Diabetes	6	Yes	Yes	No	aOR	1.34	1.28–1.41	Yes
Kurdyak et al. [45]	2017	Diabetes T1/T2	Schizophrenia	ED	All cause^a^	6	Yes	Yes	No	aOR	1.72	1.68–1.77	Yes
Kurdyak et al. [45]	2017	Diabetes T1/T2	Schizophrenia	Inpatient	Diabetes	6	Yes	Yes	No	aOR	1.36	1.28–1.43	Yes
Kurdyak et al. [45]	2017	Diabetes T1/T2	Schizophrenia	Inpatient	All cause^a^	6	Yes	Yes	No	aOR	1.85	1.79–1.92	Yes
Helmer et al. [30]	2020	Diabetes T1/T2	SMI	Inpatient	ACSC	7	Yes	Yes	No	aOR	1.00	0.94–1.07	Yes
Chwastiak et al. [42]	2014	Diabetes T1/T2	SMI	30-day	All cause^a^	8	Yes	Yes	Yes	aOR	1.24	1.07–1.44	Yes
Wang et al. [78]	2021	Diabetes T2	SMI	Inpatient	All cause^a^	9	Yes	Yes	Yes	aOR	1.36	1.13–1.65	Yes
Cramer et al. [38]	2010	Diabetes T1/T2	Psychosis	Inpatient	All cause	5	No	Yes	No	aOR	2.15	1.18–3.92	Yes, but also excluded as does not adjusted for age and sex
Helmer et al. [30]	2020	Diabetes T1/T2	SMI	Inpatient	Chronic ACSC	7	Yes	Yes	No	aOR	0.88	0.82–0.96	No: subset of all ACSC
Helmer et al. [30]	2020	Diabetes T1/T2	SMI	Inpatient	Acute ACSC	7	Yes	Yes	No	aOR	1.21	1.11–1.31	No: subset of all ACSC
Egglefield et al. [29]	2020	Diabetes T1/T2	Schizophrenia	Inpatient	Diabetes	4	No	No	No	OR^d^	1.69	1.54–1.86	No: unadjusted
Krein et al. [44]	2006	Diabetes T1/T2	SMI	Inpatient	All cause	4	No	No	No	OR	2.80	2.67–2.94	No: unadjusted
Shim et al. [46]	2014	Diabetes T1/T2	Schizophrenia	ED	Diabetes	4	No	No	No	OR	1.17	1.12–1.21	No: unadjusted
Shim et al. [46]	2014	Diabetes T1/T2	Schizophrenia	ED	All cause^a^	4	No	No	No	OR^d^	1.30	1.25–1.34	No: unadjusted
Tsai et al. [32]	2019	Diabetes T1/T2	Bipolar	Inpatient	Diabetes	8	Yes	Yes	No	aHR	1.41	1.15–1.71	Yes
Mai et al. [37]	2011	Diabetes T1/T2	Affective psychosis	Inpatient	Diabetes	8	Yes	Yes	No	aHR^e^	1.22	1.15–1.30	Yes
Edwards et al. [34]	2014	Diabetes T1/T2	Psychosis	Inpatient	ACSC	6	Yes	Yes	No	aHR	1.01	0.98–1.04	Yes
Mai et al. [37]	2011	Diabetes T1/T2	Other psychosis	Inpatient	Diabetes	8	Yes	Yes	No	aHR^e^	1.18	1.10–1.27	Yes
Mai et al. [37]	2011	Diabetes T1/T2	Schizophrenia	Inpatient	Diabetes	8	Yes	Yes	No	aHR^e^	1.06	0.94–1.20	Yes
Becker et al. [43]	2011	Diabetes T1/T2	Schizophrenia	Inpatient or ED	Diabetes	8	Yes	Yes	Yes	aHR	1.68	1.34–2.10	Yes
Chwastiak et al. [42]	2014	Diabetes T1/T2	SMI	Inpatient	All cause^a^	7	Yes	Yes	Yes	aHR	1.14	1.05–1.23	Yes
Goueslard et al. [33]	2018	Diabetes T1	Schizophrenia	Inpatient	Diabetes	6	Yes	Yes	No	aHR	2.13	1.69–2.69	Yes, but also excluded as an outlier
Stockbridge et al. [31]	2019	Diabetes T1/T2	Bipolar	Inpatient	Diabetes	7	Yes	Yes	No	aRR	1.34	0.78–2.31	No: RR
Stockbridge et al. [31]	2019	Diabetes T1/T2	Schizophrenia	Inpatient	Diabetes	7	Yes	Yes	No	aRR	1.41	0.94–2.12	No: RR
Huang et al. [73]	2021	Diabetes T2	Schizophrenia	Inpatient	All cause^a^	7	No	No	No	Average number of admissions	1.09 vs 0.92	p = 0.001	No: Average utilisation
Sullivan et al. [47]	2006	Diabetes T1/T2	SMI	Admission ratio	Diabetes	6	Yes	No	No	aOR	0.77	0.45–1.33	No: Admission ratio
**The effect of SMI on hospital utilisation in patients with cardiovascular disease**													
Shah et al. [51]	2018	Cardiogenic shock (no AMI)	Psychosis	30-day	All cause	8	Yes	Yes	No	aOR	0.90	0.78–1.05	Yes
Pham et al. [52]	2019	Heart failure	Psychosis	30-day	All cause	7	Yes	Yes	No	aOR	1.11	1.04–1.18	Yes
Pham et al. [52]	2019	Heart failure	Psychosis	30-day	Cardiovascular	7	Yes	Yes	No	aOR	1.02	0.93–1.13	Yes
Chamberlain et al. [53]	2018	Congestive heart failure	Psychosis	30-day	Cardiovascular	8	Yes	Yes	No	aOR	1.07	1.01–1.12	Yes
Chamberlain et al. [53]	2018	Congestive heart failure	Psychosis	30-day	Cardiovascular	8	Yes	Yes	No	aOR	1.08	1.00–1.16	Yes
Shah et al. [54]	2018	Takotsubo cardiomyopathy	Psychosis	30-day	All cause	8	Yes	Yes	No	aOR	1.90	1.36–2.66	Yes
Shah et al. [55]	2018	Cardiogenic shock (with AMI)	Psychosis	30-day	All cause	8	Yes	Yes	No	aOR	1.14	0.97–1.35	Yes
Coffey et al. [58]	2012	Congestive heart failure	Psychosis	30-day	Cardiovascular	7	Yes	Yes	No	aOR	1.16	p<0.001	Yes
Jorgensen et al. [56]	2017	Heart failure	Schizophrenia	30-day	All cause^a^	9	Yes	Yes	No	aOR	1.77	0.79–3.92	Yes
Ghani et al. [72]	2021	Vascular surgery	SMI	30-day	All cause^c^	6	Yes	No	Yes	aOR	2.02	1.10–3.70	Yes
Paredes et al. [75]	2020	CABG surgery	SMI	30-day	All cause	7	Yes	Yes	No	aOR^e^	2.28	2.10–2.46	Yes
Pham et al. [52]	2019	Heart failure	Psychosis	7-day	All cause	7	Yes	Yes	No	aOR	1.10	1.00–1.22	No: 7-day readmission
Pham et al. [52]	2019	Heart failure	Psychosis	7-day	Cardiovascular	7	Yes	Yes	No	aOR	1.04	0.87–1.23	No: 7-day readmission
Ahmedani et al. [57]	2015	Heart failure	Schizophrenia	30-day	All cause	6	No	No	No	OR^d^	1.06	0.78–1.44	No: unadjusted
Ahmedani et al. [57]	2015	MI	Schizophrenia	30-day	All cause	6	No	No	No	OR^d^	1.55	0.69–3.45	No: unadjusted
Ahmedani et al. [57]	2015	Heart failure	Bipolar	30-day	All cause	6	No	No	No	OR^d^	1.25	1.05–1.50	No: unadjusted
Ahmedani et al. [57]	2015	MI	Bipolar	30-day	All cause	6	No	No	No	OR^d^	0.98	0.61–1.58	No: unadjusted
Ahmedani et al. [57]	2015	Heart failure	Other psychoses	30-day	All cause	6	No	No	No	OR^d^	1.70	1.40–2.07	No: unadjusted
Andres et al. [77]	2012	MI	Schizophrenia	Inpatient	MI	6	No	No	No	OR^d^	0.83	0.25–2.81	No: unadjusted
Sreenivasan et al. [76]	2022	MI	Psychosis	30-day	All cause	8	Yes	Yes	No	aHR	1.56	1.43–1.69	Yes
Lu et al. [59]	2017	Heart failure	Bipolar	Inpatient	Cardiovascular	6	Yes	Yes	No	aHR	2.08	1.05–4.11	Yes, but also excluded as an outlier
Fleetwood et al. [71]	2021	MI	Bipolar	Inpatient	MI or stroke	8	Yes	No	No	aHR	1.40	1.20–1.62	Yes
Fleetwood et al. [70]	2021	Stroke	Bipolar	Inpatient	MI or stroke	8	Yes	No	No	aHR	1.14	1.01–1.28	Yes
Sreenivasan et al. [76]	2022	MI	Bipolar	30-day	All cause	8	Yes	Yes	No	aHR	1.32	1.19–1.45	Yes
Lu et al. [59]	2017	Heart failure	Bipolar	30-day	Cardiovascular	7	Yes	Yes	No	aHR	3.44	1.19–10.00	Yes, but also excluded as an outlier
Attar et al. [48]	2020	MI	Schizophrenia	Inpatient	Re-infarction	8	Yes	Yes	Yes	aHR	1.29	0.77–2.13	Yes
Chamberlain et al [49]	2017	Atrial fibrillation	Schizophrenia	Inpatient	All cause	7	Yes	Yes	No	aHR	1.22	0.98–1.52	Yes
Lu et al. [59]	2017	Heart failure	Schizophrenia	Inpatient	Cardiovascular	6	Yes	Yes	No	aHR	2.33	1.51–3.61	Yes, but also excluded as an outlier
Lu et al. [59]	2017	Heart failure	Schizophrenia	30-day	Cardiovascular	7	Yes	Yes	No	aHR	4.92	2.49–9.71	Yes, but also excluded as an outlier
Fleetwood et al. [71]	2021	MI	Schizophrenia	Inpatient	MI or stroke	8	Yes	No	No	aHR	1.46	1.29–1.65	Yes
Fleetwood et al. [70]	2021	Stroke	Schizophrenia	Inpatient	MI or stroke	8	Yes	No	No	aHR	1.21	1.10–1.34	Yes
Fleetwood et al. [71]	2021	MI	Schizophrenia	Inpatient	MI	8	Yes	No	No	aHR	1.42	1.24–1.63	No: Population included in other outcome
Fleetwood et al. [71]	2021	MI	Bipolar	Inpatient	MI	8	Yes	No	No	aHR	1.34	1.13–1.58	No: Population included in other outcome
Fleetwood et al. [70]	2021	Stroke	Schizophrenia	Inpatient	Stroke	8	Yes	No	No	aHR	1.24	1.11–1.38	No: Population included in other outcome
Fleetwood et al. [70]	2021	Stroke	Bipolar	Inpatient	Stroke	8	Yes	No	No	aHR	1.17	1.03–1.32	No: Population included in other outcome
Attar et al. [48]	2020	MI	Schizophrenia	Inpatient	Stroke	8	Yes	Yes	Yes	aHR	1.72	1.00–2.98	No: Population included in other outcome
Attar et al. [48]	2020	MI	Schizophrenia	Inpatient	Heart failure	8	Yes	Yes	Yes	aHR	1.39	1.04–1.86	No: Population included in other outcome
Kallio et al. [69]	2022	Coronary artery disease and CABG	Schizophrenia	Inpatient	MI	6	No	No	No	HR	1.86	1.25–2.78	No: unadjusted
Kallio et al. [69]	2022	Coronary artery disease and CABG	Schizophrenia	Inpatient	Stroke	6	No	No	No	HR	0.91	0.50–1.66	No: unadjusted
Sayers et al. [50]	2007	Heart failure	Psychosis	Inpatient	All cause	7	Yes	Yes	No	Predicted increase	0.30	p<0.001	No: predicted increase
Sayers et al. [50]	2007	Heart failure	Bipolar	Inpatient	All cause	7	Yes	Yes	No	Predicted increase	0.38	p = 0.001	No: predicted increase
Davydow et al. [66]	2016	MI	SMI	Inpatient	ACSC	5	No	No	No	RR^d^	1.41	1.36–1.47	No: RR
Davydow et al. [66]	2016	CHF	SMI	Inpatient	ACSC	5	No	No	No	RR^d^	1.19	1.15–1.22	No: RR
Davydow et al. [66]	2016	Cerebrovascular disease	SMI	Inpatient	ACSC	5	No	No	No	RR^d^	1.47	1.43–1.52	No: RR
**The effect of SMI on hospital utilisation in patients with COPD**													
Lau et al. [62]	2017	COPD	Psychosis	30-day	COPD	8	Yes	Yes	No	aOR	1.19	1.13–1.25	Yes
Lau et al. [62]	2017	COPD	Psychosis	30-day	COPD	8	Yes	Yes	No	aOR	1.16	1.08–1.24	Yes
Singh et al. [63]	2016	COPD	Psychosis	30-day	All cause	6	Yes	No	No	aOR	1.18	1.10–1.27	Yes
Jorgensen et al. [61]	2018	COPD	Schizophrenia	30-day	All cause	8	Yes	Yes	No	aOR	1.08	0.92–1.28	Yes
Buhr et al. [60]	2019	COPD	Psychosis	30-day	All cause	5	No	No	No	OR^d^	1.27	1.25–1.29	No: unadjusted
**The effect of SMI on inpatient admissions in liver disease patients**													
Huckans et al. [65]	2010	HCV	Schizophrenia	Inpatient	All cause^a^	5	No	No	No	OR	5.80	0.63–53.01	No: unadjusted
Huckans et al. [65]	2010	HCV	Schizophrenia	ED	All cause^a^	5	No	No	No	OR	3.27	0.77–13.83	No: unadjusted
Davydow et al. [66]	2016	Liver disease	SMI	Inpatient	ACSC	5	No	No	No	RR^d^	1.53	1.45–1.61	No: unadjusted
**The effect of SMI on inpatient admissions in cancer patients**													
Davydow et al. [66]	2016	Cancer	SMI	Inpatient	ACSC	5	No	No	No	RR^d^	1.54	1.48–1.60	No: unadjusted
Basta et al. [64]	2016	Breast cancer related mastectomy/ lumpectomy	Psychosis	Inpatient	Cancer	8	No^b^	Yes	No	aOR	2.15	1.51–3.06	No: limited comparison
Kashyap et al. [68]	2021	Gastrointestinal malignancies	Bipolar	ED	All cause end of life	8	Yes	Yes	No	aOR	1.12	1.01–1.24	No: limited comparison
Kashyap et al. [68]	2021	Gastrointestinal malignancies	Psychosis	ED	All cause end of life	8	Yes	Yes	No	aOR	0.98	0.85–1.12	No: limited comparison
Ratcliff et al. [74]	2021	Surgery for colorectal cancer	Bipolar	90-day	All cause	6	No	No	No	OR^d^	1.24	1.04–1.47	No: unadjusted
Ratcliff et al. [74]	2021	Surgery for colorectal cancer	Psychosis	90-day	All cause	6	No	No	No	OR^d^	1.25	1.03–1.52	No: unadjusted

a: Excluded psychiatric hospitalisations

b: Adjusted for age and only included females so scored as if adjusted for age and sex

c: emergency admissions

d: calculated from raw data

e: extracted from figure using ImageJ: https://imagej.nih.gov/ij/; COPD: Chronic Obstructive Pulmonary Disease; ED: Emergency Department; OR: odds ratio; HR: hazard ratio; RR: risk ratio; HCV: hepatitis C virus; SMI: severe mental illness; CABG: coronary artery bypass graft; MI: myocardial infarction; ACSC: ambulatory care sensitive condition.

### Study quality and risk of bias

The majority of studies had pre-existing psychiatric illness as a focus (n = 37/50), while 11 considered a broad range of risk factors for hospital admission, of which SMI was one. Two studies included SMI as a covariate for a different exposure of interest. The majority (n = 42) of studies were in unmatched populations and 11 did not provide adjusted effect measures. Ten studies were limited to a single region of a country, and two to single hospitals (S1 Table). Denominator populations were sourced from hospital records in 31 studies, hospital and outpatient or pharmacy records in eleven and primary care records in eight (S1 Table).

Of the 39 studies which provided adjusted effect estimates, 37 controlled for age and gender, one controlled for gender but not age [38] and one controlled for age and was limited to the female population only [64]. Thirty-three studies controlled for physical health comorbidities and eight for prior healthcare utilisation (S1 Table). Almost half the studies (n = 24/50) had a NOS of between 6 and 7 (fair quality), while 19 had a score of 8 or 9 (high quality) and seven had a score of under 6 (poor quality; S1 and S2 Tables). Two studies with multiple analyses had differing NOS for analyses presenting ORs and HRs (S1 and S2 Tables). Funnel plots for all analyses presenting ORs (Egger’s test: p = 0.3733, S1 Fig) and risk ratios (Egger’s test: p = 0.2809, S1 Fig) were not suggestive of publication bias, however the funnel plot for analyses presenting HRs was asymmetrical (Egger’s test: p<0.0001, S1 Fig).

### Hospital utilisation in people with SMI, comparing people with or without physical LTCs

Nine analyses from three studies [39, 46, 67] investigated the impact of diabetes (n = 5), cardiovascular disease (n = 2) and COPD (n = 2) on hospitalisation in a patient population with pre-existing schizophrenia (n = 2) or bipolar disorder (n = 7). The outcome was all-cause ED attendances for four studies and all-cause admissions for five. All analyses found a higher risk of hospital utilisation in those with SMI and a physical health condition compared to those with SMI alone (Table 2). The low number and heterogenous study characteristics meant that these studies were deemed unsuitable for meta-analysis.

### Hospital utilisation in people with physical LTCs, comparing people with and without SMI

Ninety-five analyses from 48 studies investigated the impact of SMI diagnosis on hospital utilisation in a patient population with diagnoses of diabetes, cardiovascular disease, COPD, liver disease or cancer.

#### Hospital utilisation in people with diabetes, with and without SMI

Thirty-seven analyses from 20 studies investigated the effect of SMI on hospital utilisation in patients with diabetes. Most analyses included patients diagnosed with either type I or II diabetes mellitus (n = 28; Table 2). Twenty-seven analyses were included in meta-analysis, reasons for exclusions are detailed in Table 2.

**Fig 2:**
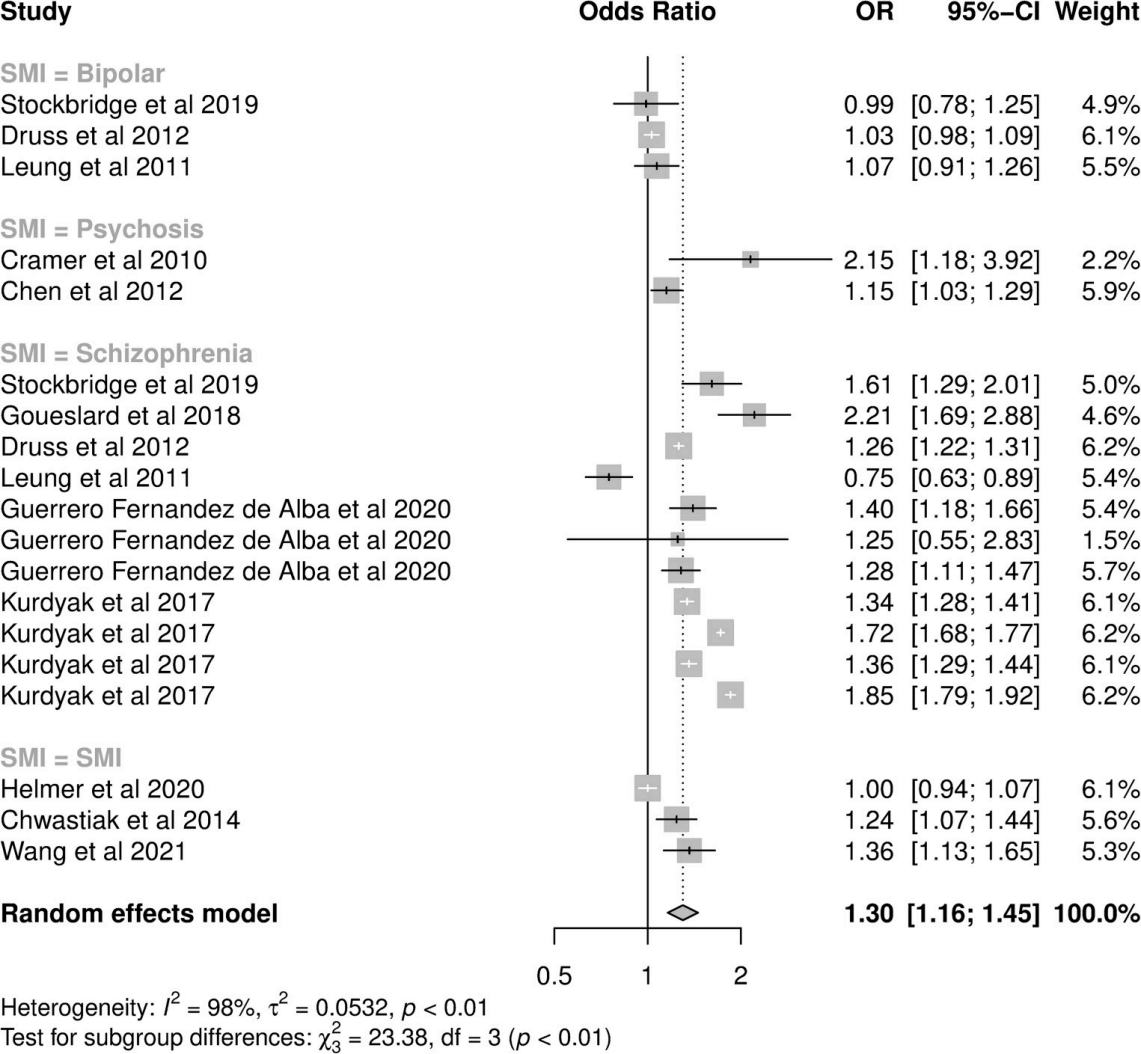
Forest plot of studies presenting adjusted odds ratios of hospital utilisation in diabetes patients with SMI compared to diabetes patients without SMI.

The meta-analysis of adjusted OR included 19 analyses from 14 studies (Fig 2). Schizophrenia was the most frequent exposure (11 analyses) and admissions the most frequent outcome (14 analyses; Table 2). The funnel plot of these analyses did not show asymmetry (Egger’s test: p = 0.0738, S2 Fig). For patients with diabetes, the pooled OR for hospital utilisation in patients with a diagnosis of any SMI was 1.30 (95%CI: 1.16–1.45) compared to those without an SMI diagnosis, however heterogeneity was high (I^2^ = 97.8%). When one study which did not control for age was removed [38] the pooled odds ratio was 1.28 (95% confidence interval (CI) 1.15–1.44, I^2^ = 97.9%) In subgroup analysis, the effect size was greater in patients with schizophrenia (OR: 1.42, 95%CI: 1.25–1.60) than patients with other SMI diagnoses, and analyses of all-cause hospitalisations had higher pooled OR (1.43, 95%CI: 1.28–1.60) compared to those reporting ACSC conditions or diabetes-specific hospitalisations (Table 3). Studies performed in the US had a lower pooled OR (1.10, 95%CI: 0.99–1.22) than studies in other countries (Table 3). While the pooled OR for analyses of 30-day readmissions was lower, confidence intervals of all outcome types overlapped (Table 3). Controlling for these variables in meta-regression reduced heterogeneity (I^2^ = 82.8%).

**Table 3:** Subgroup analyses of studies of hospital use in people with underlying diabetes, cardiovascular disease and COPD: Comparing those with and without SMI with outliers removed.

			No. of studies	Pooled effect size (95%CI) of hospital use in people with SMI compared to those without	I^2^ (%)	p-value for between group differences
**The effect of SMI on hospital use in people with diabetes (OR)**						
	SMI diagnosis					<0.0001
		Bipolar disorder	3	1.03 (0.98–1.08)	0	
		Psychosis	1	1.15 (1.03–1.29)	--	
		Schizophrenia	11	1.42 (1.25–1.60)	97.7	
		SMI	3	1.17 (0.96–1.44)	85.9	
	Outcome: service					0.2015
		30-day readmission	2	1.18 (1.08–1.29)	0	
		ED attendance	3	1.44 (1.18–1.77)	97.8	
		Inpatient admissions	13	1.26 (1.08–1.47)	97.9	
	Outcome: Cause					0.0225
		All-cause	7	1.43 (1.28–1.60)	94.7	
		Diabetes	8	1.25 (1.08–1.44)	90.3	
		Ambulatory care sensitive	3	1.09 (0.93–1.28)	96.6	
	Country of study					<0.0001
		US	9	1.10 (0.99–1.22)	91.6	
		Canada	4	1.55 (1.34–1.80)	98.2	
		France	1	2.21 (1.69–2.89)	--	
		Spain	3	1.33 (1.19–1.48)	0	
		UK	1	1.36 (1.13–1.65)	--	
**The effect of SMI on hospital use in people with diabetes (HR)**						
	SMI diagnosis					0.3654
		Bipolar disorder	2	1.27 (1.12–1.44)	46.2	
		Psychosis	2	1.09 (0.93–1.27)	93.2	
		Schizophrenia	2	1.32 (0.85–2.07)	91.8	
		SMI	1	1.14 (1.05–1.23)	--	
	Outcome: Cause					<0.0001
		All-cause	1	1.14 (1.05–1.23)	--	
		Diabetes	5	1.25 (1.13–1.37)	73.5	
		Ambulatory care sensitive	1	1.01 (0.98–1.04)	--	
	Country of study					0.0016
		US	2	1.07 (0.95–1.20)	87.3	
		Canada	1	1.68 (1.34–2.10)	--	
		Australia	3	1.17 (1.10–1.25)	46.5	
		Taiwan	1	1.41 (1.16–1.72)	--	
**The effect of SMI on hospital use in people with cardiovascular disease (OR)**						
	SMI diagnosis					<0.0001
		Psychosis	8	1.09 (1.02–1.16)	66.4	
		Schizophrenia	1	1.77 (0.79–3.94)	--	
		SMI	2	2.28 (2.11–2.46)	0	
	Outcome: Cause					0.0861
		All-cause	7	1.46 (1.03–2.08)	97.5	
		Cardiovascular disease	4	1.07 (1.04–1.11)	0	
	Country of study					0.2259
		US	9	1.22 (1.01–1.48)	97.5	
		Denmark	1	1.77 (0.79–3.94)	--	
		UK	1	2.02 (1.10–3.70)	--	
**The effect of SMI on hospital use in people with cardiovascular disease (HR)**						
	SMI diagnosis					0.0056
		Bipolar	3	1.28 (1.13–1.43)	62.9	
		Psychosis	1	1.56 (1.44–1.67)	--	
		Schizophrenia	4	1.30 (1.15–1.46)	47.8	
	Outcome: Service					0.2218
		30-day readmission	2	1.44 (1.22–1.69)	53.7	
		Inpatient admissions	6	1.28 (1.17–1.40)	84.4	
	Outcome: Cause					0.4218
		All-cause	3	1.39 (1.20–1.60)	77.0	
		Cardiovascular disease	5	1.29 (1.16–1.43)	62.5	
	Country of study					0.7365
		Sweden	1	1.29 (0.78–2.14)	--	
		UK	4	1.29 (1.15–1.44)	71.8	
		US	3	1.39 (1.20–1.60)	77.0	
**The effect of SMI on hospital use in people with COPD (OR)**						
	SMI diagnosis					0.3059
		Psychosis	3	1.18 (1.14–1.22)	0	
		Schizophrenia	1	1.08 (0.92–1.27)	--	
	Outcome: Cause					0.7298
		All-cause	2	1.16 (1.09–1.24)	0	
		COPD	2	1.18 (1.13–1.23)	0	
	Country of study					0.3059
		US	3	1.18 (1.14–1.22)	0	
		Denmark	1	1.08 (0.92–1.27)	--	

**Fig 3:**
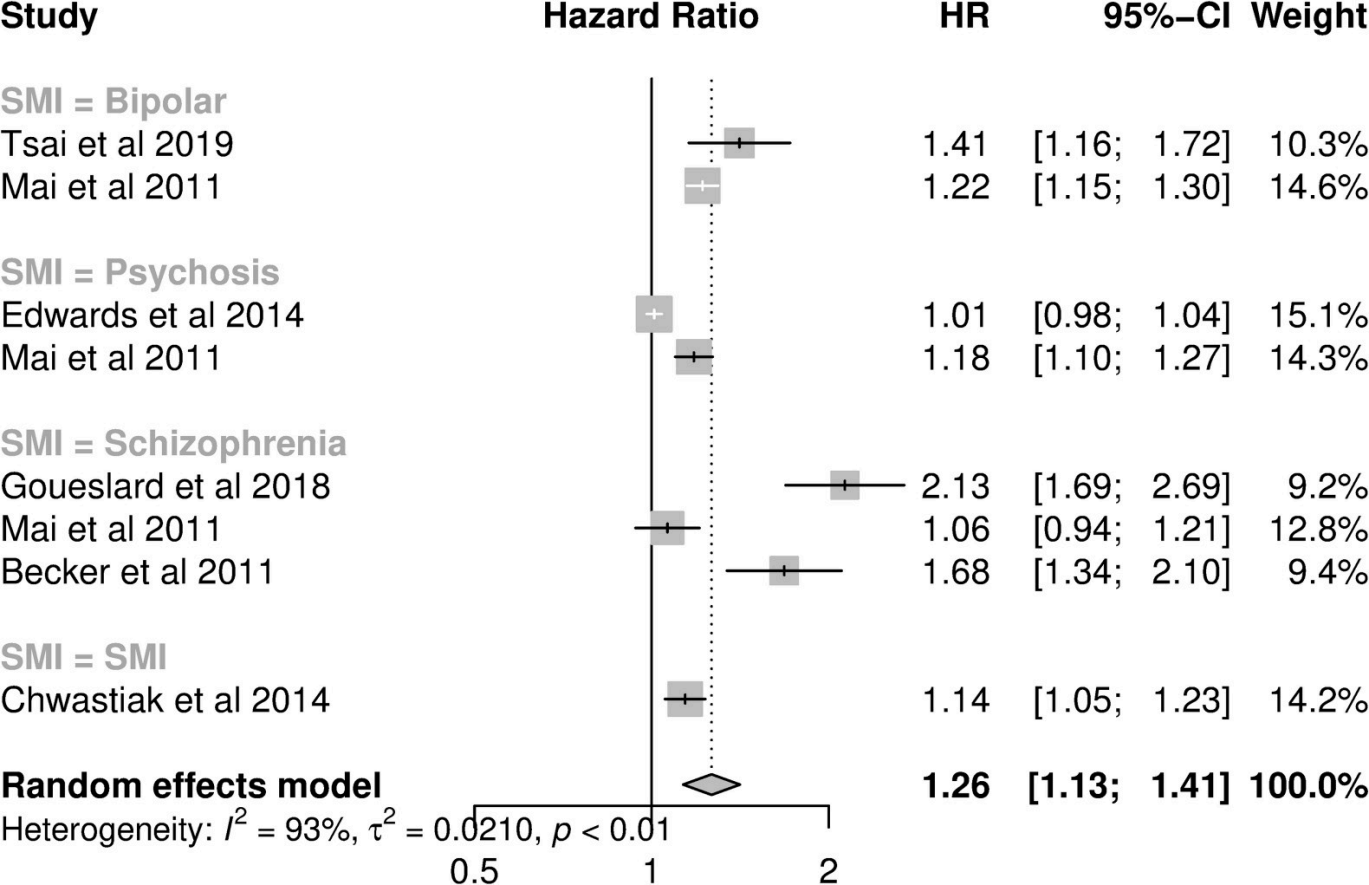
Forest plot of studies presenting adjusted hazard ratios of hospital utilisation in diabetes patients with SMI compared to diabetes patients without SMI.

Fewer studies in populations with diabetes assessed HR (eight analyses from six studies, Fig 3). Seven analyses investigated admissions, while one investigated admissions or ED attendance combined (Table 2). The funnel plot identified one outlier, with a large effect size [33] (S3 Fig). When this outlier was removed, the pooled HR was reduced from 1.26 (1.13–1.41; I^2^ = 92.7%, Fig 3) to 1.19 (95%CI: 1.08–1.31, I^2^ = 90.6%). In subgroup analysis, analyses of diabetes admissions had a higher pooled HR (1.25; 95%CI: 1.13–1.37) than all-cause or ACSC admissions studies, while analyses performed in the US had a lower pooled HR (1.07; 95%CI: 0.95–1.20) than studies in other countries (Table 3). Pooled HRs were similar across SMI diagnoses. When controlling for country and type of hospital utilisation in meta-regression, the residual heterogeneity was reduced (I^2^: 46.5%).

For studies of both hazard ratios and odds ratios based in the US, there was evidence that pooled effect sizes of hospital utilisation in people with SMI were lower in studies of patients registered in Veteran’s Affairs, Medicare or Medicaid, compared to studies of commercially insured people or studies including both state-insured and commercially insured individuals (Table 4).

**Table 4:** Subgroup analyses of studies of hospital use in the US in people with underlying diabetes: Comparing those with and without SMI.

			No. of studies	Pooled effect size (95%CI) of hospital use in people with SMI compared to those without	I^2^ (%)	p-value for between group differences
**The effect of SMI on hospital use in people with diabetes (OR)**						
	Study population					0.0365
		Medicaid/Medicare	4	1.03 (0.86–1.22)	95.4	
		Veterans’ health	1	1.00 (0.94–1.07)	--	
		Insured	3	1.22 (0.96–1.56)	80.1	
		Complete	1	1.24 (1.07–1.44)	--	
**The effect of SMI on hospital use in people with diabetes (HR)**						
	Study population					0.005
		Veterans’ health	1	1.01 (0.98–1.04)		
		Insured	1	1.14 (1.05–1.23)		

#### Hospitalisation use in people with cardiovascular disease, with and without SMI

Forty-four analyses from 20 studies were based in populations with underlying cardiovascular disease, the most common of which was heart failure (n = 7, Table 1). Eleven analyses from nine studies providing adjusted ORs for hospital utilisation in people with SMI compared to those without SMI were included in meta-analysis, and twelve analyses from six studies presented adjusted HR. The funnel plot for these analyses did not show asymmetry (meta-analysis of ORs: Egger’s test: p = 0.6751, S4 Fig; meta-analysis of HRs: Egger’s test: p = 0.1535, S5 Fig), and for ORs did not show any outliers.

**Fig 4:**
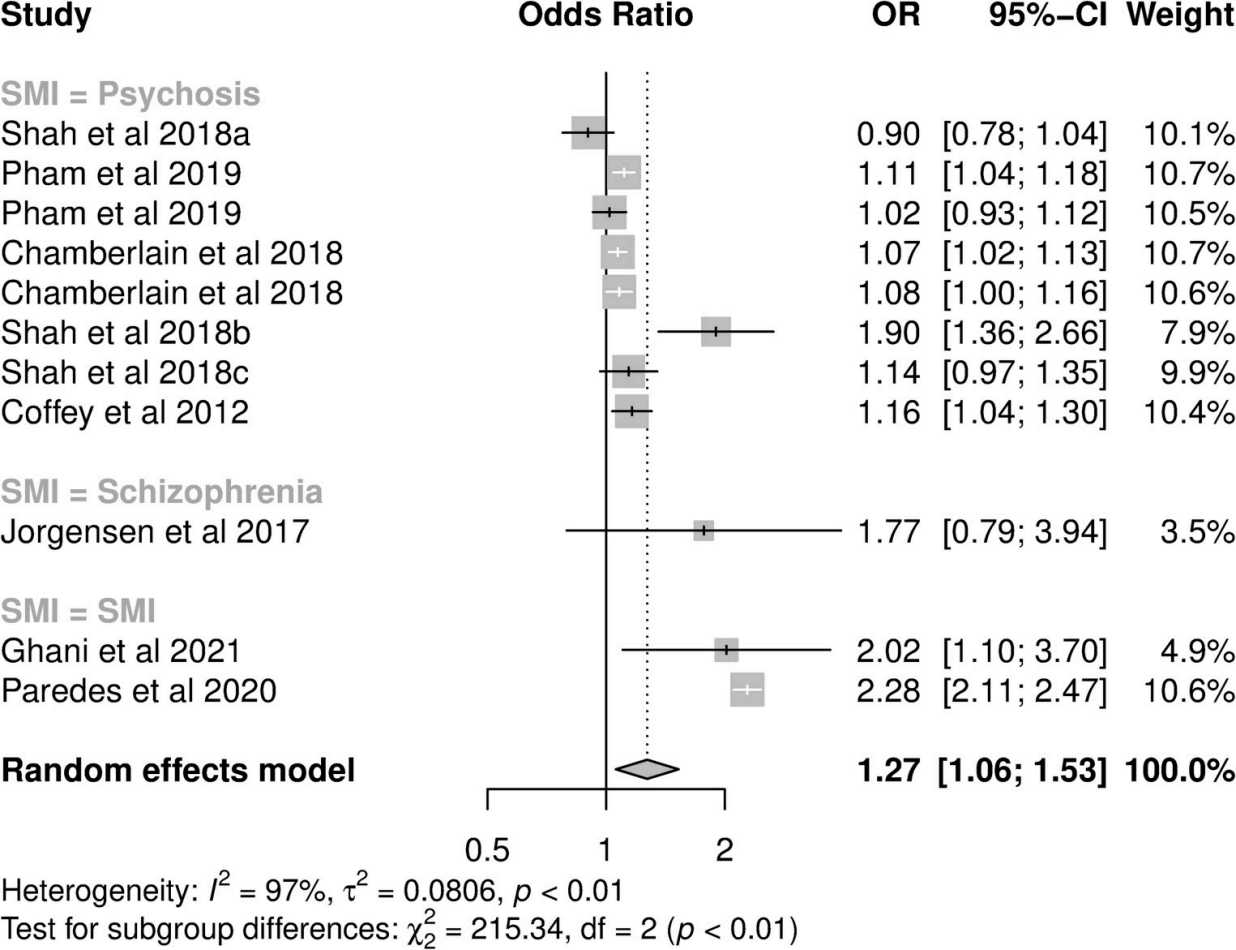
Forest plot of studies presenting adjusted odds ratios of hospital utilisation in cardiovascular disease patients with SMI compared to cardiovascular disease without SMI. a: [51], b: [54], c: [55].

For those presenting ORs, all were 30-day readmission studies, and psychosis was the exposure for eight analyses (Table 2). The pooled OR for hospital utilisation in patients with a diagnosis of any SMI was 1.27 (95%CI: 1.06–1.53; I^2^: 96.9%, Fig 4). In subgroup analysis, pooled OR were not significantly different between cause of hospitalisation or country of study, but did differ by SMI diagnosis (Table 3). The majority of analyses examined broad risk factors for hospitalisation, while only three focused on SMI specifically. Those with SMI as a focus had greater pooled OR (pOR: 2.27, 95%CI: 2.10–2.46 vs. pOR 1.09, 95%CI: 1.02–1.16). Controlling for these variables in meta-regression reduced heterogeneity (I^2^ = 61.9%).

**Fig 5:**
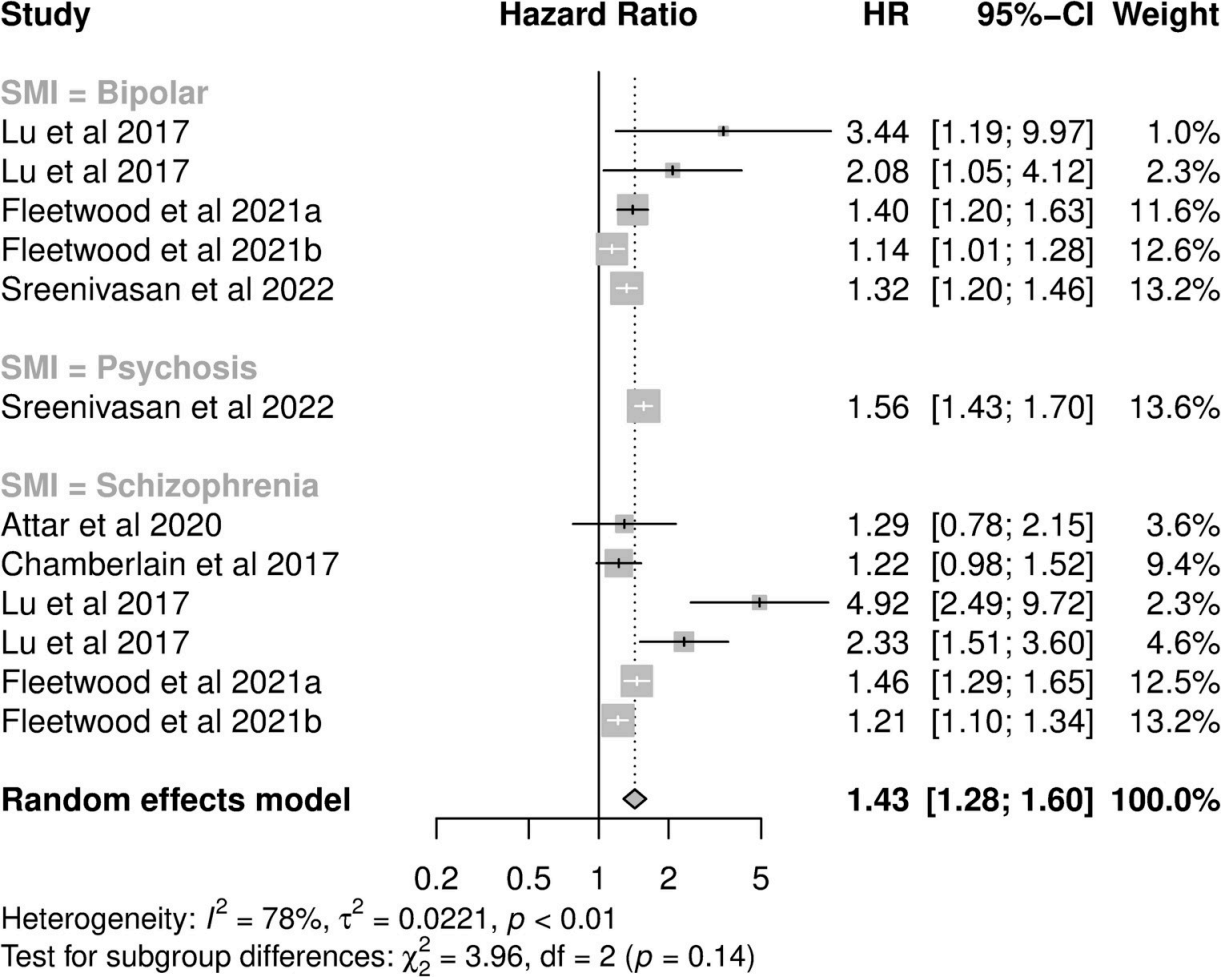
Forest plot of studies presenting adjusted hazard ratios of hospital utilisation in cardiovascular disease patients with SMI compared to cardiovascular disease without SMI. a: [71] b: [70].

For those presenting HRs, the pooled HR for hospital utilisation was 1.43 (95%CI: 1.28–1.60, I^2^: 78.4%, Fig 5). Most analyses investigated inpatient admissions (8/12) and cardiovascular outcomes (n = 9). One study, contributing four analyses, was identified as an outlier (S5 Fig). This study was a small single-site study of African American patients in the US [59]. Removal of this study from the meta-analysis reduced the pooled HR to 1.33 (95%CI: 1.21–1.46, I^2^: 74.0%). In subgroup analysis, pooled HRs were not significantly different between cause of hospitalisation, hospitalisation type or country of study (Table 3). However, there were differences by SMI diagnosis, and controlling for this did reduce heterogeneity (I^2^ = 55.13%).

#### Hospitalisation use in people with COPD, with and without SMI

**Fig 6:**
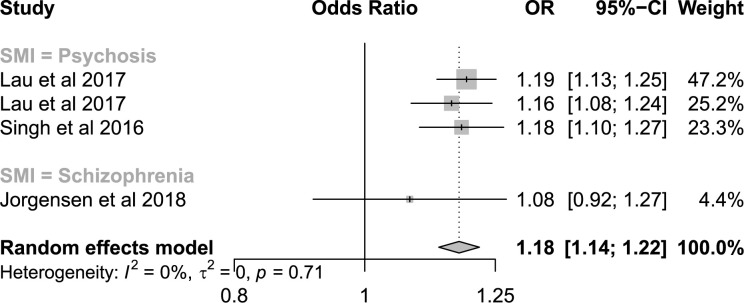
Forest plot of studies presenting adjusted odds ratios of hospital utilisation in COPD patients with SMI compared to COPD patients without SMI.

Five analyses from four studies were in populations with underlying COPD. All five presented ORs for 30-day readmissions in patients with SMI compared to those without SMI, of which four presented adjusted ORs. The funnel plot of these analyses did not show asymmetry of outliers (S6 Fig). The pooled OR for hospital use in patients with a diagnosis of any SMI was 1.18 (95%CI: 1.14–1.22, I^2^ = 0%, Fig 6). In subgroup analysis, pooled ORs were not significantly different between cause of hospitalisation, country of study or SMI diagnosis (Table 3).

#### Hospitalisation use in people with cancer or liver disease, with and without SMI

Two studies were identified which considered SMI as an exposure for hospitalisation in people with and without SMI in populations with underlying liver disease and four in populations with underlying cancer (Table 1). Neither of the liver disease studies presented adjusted effect estimates, and both were low quality for the exposures and outcomes considered in this synthesis (NOS score = 5). Huckans et al. [65] found that people with schizophrenia were more likely to attend EDs and have inpatient admissions during hepatitis C treatment than those without schizophrenia, though due to the small population size (n = 60) confidence intervals were wide and included one. Davydow et al. [66] found higher ACSC admissions for in those with liver disease and SMI compared to those with liver disease without SMI (Table 2).

For cancer, two studies presented adjusted effect measures of hospital utilisation. Basta et al. [64] studied readmissions for lymphedema in the two years after breast cancer diagnosis in women. They found that women with a diagnosis of psychosis were at higher risk of readmission (aOR: 2.15, 95%CI: 1.51–3.06). Kashyap et al. [68] found higher utilisation of emergency departments in the 30 days prior to death in those with gastrointestinal malignancies and SMI compared to those with gastrointestinal malignancies alone. Finally, an unadjusted analysis by Ratcliff et al. [74] found higher risk of 90-day readmissions after surgery for colorectal cancer in those with SMI, while Davydow et al. [66] found higher risk of ACSC admissions in those with cancer and SMI compared to those with cancer alone, in unadjusted analysis (Table 2).

*Sensitivity analysis*. In sensitivity analysis, re-running the analysis as a three-level hierarchical model did not result in improved model fit, nor substantial change the pooled OR (1.26, 95%CI: 1.10–1.45) or HR (1.23; 95%CI 1.01–1.50) for studies in people with diabetes and SMI, or the pooled OR (1.34, 95%CI: 1.07–1.69) or HR (1.47. 95%CI: 1.16–1.85) for people with cardiovascular disease and SMI. For COPD, the two analyses from one study included in the meta-analysis were from different populations and so sensitivity analysis was not performed. Only three conference abstracts providing adjusted effect measures for hospitalisation were retrieved. The first was a study of risk factors for 30- and 90-day rehospitalisation following radical cystectomy for bladder cancer. The authors found that people with psychosis had an elevated HR for readmission (aHR: 1.82, p<0.05) [79]. The second was a small study of 373 people with diabetes, which found that those with two or more admissions were more likely to have a diagnosis of schizophrenia (aOR: 4.99, p<0.05) than those with only one admission [80]. Finally, a study of all-cause 30-day readmissions in people with acute ischaemic stroke, found those with SMI we at higher risk (aOR: 1.24, 95%CI: 1.20–1.27) [81].

## Discussion

This review and meta-analysis demonstrates that people with SMI and one of five physical health conditions have consistently higher hospital utilisation than either people with SMI alone or with physical health conditions alone. This is the first systematic review to consider the impact of having SMI and a specific physical health condition on hospital utilisation, allowing a better understanding of the impact of SMI on hospital use in those with underlying physical illness, and highlighting areas for future research.

We found that in people with underlying cardiovascular disease, COPD or diabetes, people with a diagnosis of SMI had higher hospital use compared to those without SMI. This finding is in line with other systematic reviews or meta-analyses [11–13, 17], which consider the impact of SMI on hospitalisations in the general population, or when controlling for physical health comorbidities. The same appeared to be true for people with cancer and liver disease, though studies presenting adjusted analyses were limited to one study of breast cancer complications [64], and one of end of life emergency department use in people with gastrointestinal malignancies [68]. No studies of liver disease reported adjusted effect measures. Only five studies were identified which considered a population with underlying severe mental illness, with and without physical LTCs. In these studies, the addition of physical LTC increased the risk of hospital utilisation.

In populations with underlying diabetes, cardiovascular disease and COPD, people with SMI were at higher risk of 30-day readmissions compared to those without SMI, and the pooled OR were similar for 30-day readmission in these populations. This suggests that over this short timeframe, the risk of readmission does not differ substantially by underlying physical disease. While the effect size of having SMI was relatively small for all three diseases, any increased risk of hospital admission represents a major burden given the underlying high rate of admissions for these diseases in the general population [82, 83].

A strength of focusing on studies in populations with underlying physical LTC, is that it provides further evidence that the higher emergency hospitalisation in people with SMI is not due to higher prevalence of that LTC in the SMI population. It also allows the investigation of the impact of hospitalisations for the underlying LTC, compared to all-cause hospitalisations. In 30-day readmission studies of both COPD and cardiovascular disease we found little difference between studies of all-cause or cause-specific hospitalisations, suggesting that 30-day readmissions for the index condition are likely driving the difference between those with and without a diagnosis of SMI. The consistently higher risk in those with SMI, may indicate systematic differences in management and treatment of physical health conditions in people with SMI, such as lower adherence to medication, reduced access or attendance at planned outpatient care [84] and less guideline-recommended treatment [56, 61, 85–87], as well as more complex medication regimens and medical histories.

For studies examining hospital admissions for populations with underlying diabetes, we found that while patients with SMI had higher pooled OR of diabetes-specific admissions than those without SMI, the greatest difference was in all-cause admissions. This was also true in studies investigating both all-cause and diabetes admissions in the same study [41, 45]. These findings suggest that while a higher risk of diabetes admissions and sub-optimal management and treatment of diabetes [35, 37, 45] account for some of the higher hospital use in people with SMI, there are other factors involved. A study of patients with underlying diabetes found high rates of all-cause hospitalisations in people with SMI, even once acute psychiatric admissions were excluded from the outcome [45], suggesting that higher rates of multimorbidity, and therefore higher general physical health admissions, as well as higher risk of trauma and infectious disease hospitalisations [16], may be adding to the burden of hospitalisations in these patients. While we did not find the same in the subgroup analysis of diabetes studies presenting hazard ratios, only one study investigated all-cause admissions and the total number of available studies was small, limiting interpretation.

We also found evidence that specific populations may have elevated risk of hospital use. We found a high risk of hospitalisation in people with SMI in studies examining the effect of SMI on readmissions during hepatitis C treatment [65], on cardiovascular hospital use in African American patients with heart failure [59], on diabetes readmissions in patients under the age of 35 with type I diabetes [33], and following breast cancer surgery [64]. For diabetes, patients with schizophrenia appeared to be at higher risk of hospitalisation compared to other SMI diagnoses in studies presenting adjusted odds ratios. This has been reported elsewhere [17], and is in line with other studies that have found people with schizophrenia suffer more ill-health, greater all-cause mortality and poorer physical health and treatment outcomes than people with other SMI diagnoses [9, 35, 37, 88, 89]. However, for studies of people with underlying diabetes or cardiovascular disease presenting adjusted hazard ratios, there was little difference between diagnoses of bipolar disorder and schizophrenia. Of the seven studies included in our review which considered schizophrenia alongside other SMI diagnoses, two found patients with schizophrenia were more likely to be hospitalised than other SMI diagnoses [31, 35], one found that those with schizophrenia were less likely to be hospitalised [36], and four found no significant difference [37, 59, 70, 71].

Finally, we found that while still elevated, the risk of readmission in patients with diabetes and SMI was lower in the US compared to other countries. While this finding has been documented before [17], the reason for this is unclear. For these studies, we found differences in effect size based on the healthcare system under investigation, and therefore patients with SMI may face different barriers and drivers to hospital use across payers in the US healthcare system. It is not clear whether this is limited to diabetes management, as the small number of studies in patients with COPD or cardiovascular disease did not permit comparisons by country.

### Limitations

Although this review has better described the pattern of hospital utilisation in people with SMI and physical health conditions, there are limitations. Although our search strategy was thorough, we may have missed studies which include SMI as a risk factor for higher healthcare utilization, but which do not include terms for SMI in the title or abstract. These studies are unlikely to have SMI as their main exposure variable and given that SMI is not common in the general population are less likely to provide well powered estimations. We identified 11 studies for which SMI was not the main focus, and while inclusion of these studies provides further evidence, caution is needed as they may be subject to confounding and issues of power [90]. In addition, while our search strategy was thorough, and overall agreement between reviewers was high (91%), the interrater reliability of screened abstracts as measured by the Kappa statistic was moderate (0.57). This is in part due to the large number of studies screened and the rarity of relevant studies [91], but also the complexity of multiple exposures and outcomes. All disagreements were discussed thoroughly to ensure the accuracy of study inclusion.

We found marked heterogeneity in the study results, particularly for studies of diabetes. While definitions of SMI, physical LTCs and outcome measures accounted for some of this, underlying differences in the population and healthcare system, as well as differences in study design are likely major causes of this heterogeneity.

While most studies we identified were of fair or good quality, there were limitations to many of them. Few studies utilised matched cohorts of patients, and most did not evaluate the impact of prior healthcare utilisation, despite this being a known predictor of hospital use in the general population [92]. Furthermore, many studies were performed in the US, which limits the generalisability of results to other healthcare systems. Despite being based in longitudinal populations, under half of studies performed a time-to-event analysis. Where this was performed, very few accounted for multiple hospitalisations or included time-varying covariates. Most studies included only patients who had accessed secondary care, both to define SMI and physical health conditions. Without access to primary care records, these studies exclude those patients who may be managed solely in primary care or attend secondary care very infrequently. These excluded patients may provide important information on protective factors that reduce secondary care use.

### Knowledge gaps and future research

There were few studies investigating hospital use in a population of patients with SMI, comparing hospital use in those with or without physical LTC. The underlying heterogeneity of these studies made them unsuitable for meta-analysis. Given that people with SMI are at an higher risk of many physical LTCs, further research is required to identify the drivers of physical health hospitalisations in people with SMI, and subsets of this population at higher risk.

There was also a lack of data regarding hospital use in patients with cancer, and the impact of SMI diagnoses on hospital utilisation. Given the higher risk of mortality following cancer diagnosis in those with SMI, and evidence of sub-optimal cancer screening and late diagnoses [93], it is important to understand hospital utilisation in this population.

Finally, there was a lack of information on the impact of SMI on hospitalisation for liver disease, and on the long-term risk of hospitalisation in patients with COPD or cardiovascular disease. These common diseases represent a huge burden in terms of hospital resource use and ill health in the general population [94]. Given people with SMI may be at higher risk of these diseases [2], receive poorer care [6, 56, 61, 84–87, 89, 95–98] and worse outcomes [6], more research is required into the impact of an SMI diagnosis on hospital utilisation in people with these conditions.

## Conclusions

This systematic review and meta-analysis found that patients with SMI and underlying physical health conditions are at a higher risk of hospital use for that condition, and for other causes. Further research is warranted into the effects of different physical health conditions and different SMI diagnoses on hospital use, particularly over longer time periods, and of pathways and drivers of hospitalisation in those with SMI. This will allow targeted interventions aimed at reducing inappropriate hospital use and improving disease management and outcomes in people with SMI.

## Supporting information

S1 Checklist (DOC)

S1 Appendix Search strategy. (DOCX)

S1 Table Study quality and detailed characteristics. *Does control for age and is limited to females.
(DOCX)

S2 Table Components of the Newcastle-Ottawa score. *One point. a: Analysis presenting odds ratios; b: analysis presenting hazard ratios c: Analysis of 30-day readmissions; d: analysis of long-term readmissions.
(DOCX)

S1 Fig Funnel plots for all individual analyses. (DOCX)

S2 Fig Funnel plot for studies presenting adjusted odds ratios of hospital utilisation in diabetes patients with SMI compared to without SMI. (DOCX)

S3 Fig Funnel plot for studies presenting adjusted hazard ratios of hospital utilisation in diabetes patients with SMI compared to without SMI. (DOCX)

S4 Fig Funnel plot for studies presenting adjusted odds ratios of hospital utilisation in heart disease patients with SMI compared to without SMI. (DOCX)

S5 Fig Funnel plot for studies presenting adjusted hazard ratios of hospital utilisation in heart disease patients with SMI compared to without SMI. (DOCX)

S6 Fig Funnel plot for studies presenting adjusted odds ratios of hospital utilisation in COPD patients with SMI compared to without SMI. (DOCX)
