# Supplementary material for: The impact of comorbid severe mental illness and common chronic physical health conditions on hospitalisation: A systematic review and meta-analysis
Source: PLoS One. 2022 Aug 18;17(8):e0272498. doi: 10.1371/journal.pone.0272498 (PMC9387848; doi:10.1371/journal.pone.0272498)
Supplement: S1 Appendix — (DOCX) [file pone.0272498.s002.docx]

### S1 Appendix: Search strategy

**Embase**

(((severe mental illness) or (serious mental illness) or schizophrenia or (bipolar disorder)).ti,ab,kw or exp "schizophrenia spectrum disorder"/ or exp "bipolar disorder"/)

AND

((diabetes OR COPD OR (cardiovascular disease) OR cancer OR (heart disease) OR (atrial fibrillation) OR (cardiomyopathy) OR stroke OR (chronic obstructive pulmonary) OR (liver disease) OR cirrhosis OR hepatitis).ti,ab,kw or exp chronic obstructive lung disease/ or exp diabetes mellitus/ or cardiovascular disease/ or heart disease/ or vascular disease/ OR exp malignant neoplasm/ or exp cerebrovascular accident/ OR exp liver disease/)

AND

(((admission OR re-admission OR readmission OR attendance OR visit OR admitted) .ti,ab,kw

AND

((emergency department) OR "accident and emergency" OR (secondary care) OR hospital OR inpatient OR outpatient OR planned OR unplanned) .ti,ab,kw)

OR

((healthcare utili#ation) OR (health care utili#ation) OR hospitali#ation OR "length of stay" OR hospitali#ed) .ti,ab,kw)

Or

(((psychosis or psychoses or elixhauser).ti,ab)

AND

((diabetes OR COPD OR (cardiovascular disease) OR cancer OR (heart disease) OR (atrial fibrillation) OR (cardiomyopathy) OR stroke OR (chronic obstructive pulmonary) OR (liver disease) OR cirrhosis OR hepatitis).ti)

AND

(((admission OR re-admission OR readmission OR attendance OR visit OR admitted) .ti,ab,kw

AND

((emergency department) OR "accident and emergency" OR (secondary care) OR hospital OR inpatient OR outpatient OR planned OR unplanned) .ti,ab,kw)

OR

((healthcare utili#ation) OR (health care utili#ation) OR hospitali#ation OR "length of stay" OR hospitali#ed) .ti,ab,kw))

Or

(((psychosis or psychoses).ti)

AND

((diabetes OR COPD OR (cardiovascular disease) OR cancer OR (heart disease) OR (atrial fibrillation) OR (cardiomyopathy) OR stroke OR (chronic obstructive pulmonary) OR (liver disease) OR cirrhosis OR hepatitis).ti,ab,kw)

AND

(((admission OR re-admission OR readmission OR attendance OR visit OR admitted) .ti,ab,kw

AND

((emergency department) OR "accident and emergency" OR (secondary care) OR hospital OR inpatient OR outpatient OR planned OR unplanned) .ti,ab,kw)

OR

((healthcare utili#ation) OR (health care utili#ation) OR hospitali#ation OR "length of stay" OR hospitali#ed) .ti,ab,kw))

**PubMED**

(("severe mental illness" or "serious mental illness" or “schizophrenia” or “bipolar” or "schizophrenia spectrum and other psychotic disorders"[MeSH] or "bipolar and related disorders"[MeSH])

AND

(diabetes OR “cardiovascular disease” OR “cardiovascular diseases”[MeSH] OR “heart disease” OR “atrial fibrillation” OR “cardiomyopathy” OR “heart diseases”[MeSH] OR cancer OR “stroke” OR  "stroke"[MeSH Terms] OR “COPD” OR “chronic obstructive pulmonary disease” OR “hepatitis” OR “pulmonary disease, chronic obstructive”[MeSH Terms] OR “liver disease” OR “cirrhosis” OR “liver diseases”[MeSH Terms])

AND

(((“emergency department" OR "accident and emergency" OR "secondary care" OR “hospital” OR inpatient OR outpatient OR “planned” OR unplanned)

AND

(admission OR re-admission OR readmission OR “attendance” OR “attending” OR “attendances” OR “visit” OR “visits” OR admitted))

OR

("healthcare utilization" OR "health care utilization" OR "healthcare utilisation" OR "health care utilisation" OR “length of stay” OR "hospitalisation"[All Fields] OR "hospitalization"[MeSH Terms] OR "hospitalization"[All Fields] OR "hospitalised"[All Fields] OR "hospitalising"[All Fields] OR "hospitalisations"[All Fields] OR "hospitalizations"[All Fields] OR "hospitalize"[All Fields] OR "hospitalized"[All Fields])))

OR

(("psychosis"[Title/Abstract] or "psychoses"[Title/Abstract] or “elixhauser”[Title/Abstract])

AND

(diabetes[Title] OR “cardiovascular disease”[Title] OR “heart disease”[Title] OR “atrial fibrillation”[Title] OR “cardiomyopathy”[Title] OR cancer[Title] OR “stroke”[Title] OR “COPD”[Title] OR “chronic obstructive pulmonary disease”[Title] OR “hepatitis”[Title] OR “liver disease”[Title] OR “cirrhosis”[Title])

AND

(((“emergency department" OR "accident and emergency" OR "secondary care" OR “hospital” OR inpatient OR outpatient OR “planned” OR unplanned)

AND

(admission OR re-admission OR readmission OR “attendance” OR “attending” OR “attendances” OR “visit” OR “visits” OR admitted))

OR

("healthcare utilization" OR "health care utilization" OR "healthcare utilisation" OR "health care utilisation" OR “length of stay” OR "hospitalisation"[All Fields] OR "hospitalization"[MeSH Terms] OR "hospitalization"[All Fields] OR "hospitalised"[All Fields] OR "hospitalising"[All Fields] OR "hospitalisations"[All Fields] OR "hospitalizations"[All Fields] OR "hospitalize"[All Fields] OR "hospitalized"[All Fields])))

OR

(("psychosis"[Title] or "psychoses"[Title])

AND

(diabetes OR “cardiovascular disease” OR “cardiovascular diseases”[MeSH] OR “heart disease” OR “atrial fibrillation” OR “cardiomyopathy” OR “heart diseases”[MeSH] OR cancer OR “stroke” OR  "stroke"[MeSH Terms] OR “COPD” OR “chronic obstructive pulmonary disease” OR “hepatitis” OR "pulmonary disease, chronic obstructive"[MeSH Terms] OR “liver disease” OR “cirrhosis” OR "liver diseases"[MeSH Terms])

AND

(((“emergency department" OR "accident and emergency" OR "secondary care" OR “hospital” OR inpatient OR outpatient OR “planned” OR unplanned)

AND

(admission OR re-admission OR readmission OR “attendance” OR “attending” OR “attendances” OR “visit” OR “visits” OR admitted))

OR

("healthcare utilization" OR "health care utilization" OR "healthcare utilisation" OR "health care utilisation" OR “length of stay” OR "hospitalisation"[All Fields] OR "hospitalization"[MeSH Terms] OR "hospitalization"[All Fields] OR "hospitalised"[All Fields] OR "hospitalising"[All Fields] OR "hospitalisations"[All Fields] OR "hospitalizations"[All Fields] OR "hospitalize"[All Fields] OR "hospitalized"[All Fields])))

**Web of science**

((TS = ("severe mental illness" or "serious mental illness" or schizophrenia or bipolar) OR TI = ("severe mental illness" or "serious mental illness" or schizophrenia or bipolar))

AND

(TS = (diabetes OR “cardiovascular disease” OR “heart disease” OR “atrial fibrillation” OR cardiomyopathy OR “liver disease” or cirrhosis or cancer OR stroke OR COPD OR “chronic obstructive pulmonary disease” or hepatitis) OR TI=(diabetes OR “cardiovascular disease” OR heart or liver OR cancer OR stroke OR COPD OR “chronic obstructive pulmonary disease” OR hepatitis))

AND

(((TS=(“emergency department" OR "accident and emergency" OR "secondary care" OR hospital OR inpatient OR outpatient OR planned OR unplanned)

OR

TI=(“emergency department" OR "accident and emergency" OR "secondary care" OR hospital OR inpatient OR outpatient OR planned OR unplanned))

AND

(TS=(admission OR re-admission OR readmission OR attendance OR visit OR admitted)

OR

TI=(admission OR re-admission OR readmission OR attendance OR visit OR admitted)))

OR

(TS=("healthcare utilization" OR "health care utilization" OR hospitalization OR "healthcare utilisation" OR "health care utilisation" OR “length of stay” OR hospitalised OR hospitalized)

OR

TI=("healthcare utilization" OR "health care utilization" OR hospitalization OR "healthcare utilisation" OR "health care utilisation" OR “length of stay” OR hospitalised OR hospitalized))))

OR

((TS = (psychosis or psychoses or elixhauser) OR TI = (psychosis or psychoses or elixhauser))

AND

(TI=(diabetes OR “cardiovascular disease” OR heart or liver OR cancer OR stroke OR COPD OR “chronic obstructive pulmonary disease” OR hepatitis))

AND

(((TS=(“emergency department" OR "accident and emergency" OR "secondary care" OR hospital OR inpatient OR outpatient OR planned OR unplanned)

OR

TI=(“emergency department" OR "accident and emergency" OR "secondary care" OR hospital OR inpatient OR outpatient OR planned OR unplanned))

AND

(TS=(admission OR re-admission OR readmission OR attendance OR visit OR admitted)

OR

TI=(admission OR re-admission OR readmission OR attendance OR visit OR admitted)))

OR

(TS=("healthcare utilization" OR "health care utilization" OR hospitalization OR "healthcare utilisation" OR "health care utilisation" OR “length of stay” OR hospitalised OR hospitalized)

OR

TI=("healthcare utilization" OR "health care utilization" OR hospitalization OR "healthcare utilisation" OR "health care utilisation" OR “length of stay” OR hospitalised OR hospitalized))))

OR

((TI = (psychosis or psychoses))

AND

(TS = (diabetes OR “cardiovascular disease” OR “heart disease” OR “atrial fibrillation” OR cardiomyopathy OR “liver disease” or cirrhosis or cancer OR stroke OR COPD OR “chronic obstructive pulmonary disease” or hepatitis) OR TI=(diabetes OR “cardiovascular disease” OR heart or liver OR cancer OR stroke OR COPD OR “chronic obstructive pulmonary disease” OR hepatitis))

AND

(((TS=(“emergency department" OR "accident and emergency" OR "secondary care" OR hospital OR inpatient OR outpatient OR planned OR unplanned)

OR

TI=(“emergency department" OR "accident and emergency" OR "secondary care" OR hospital OR inpatient OR outpatient OR planned OR unplanned))

AND

(TS=(admission OR re-admission OR readmission OR attendance OR visit OR admitted)

OR

TI=(admission OR re-admission OR readmission OR attendance OR visit OR admitted)))

OR

(TS=("healthcare utilization" OR "health care utilization" OR hospitalization OR "healthcare utilisation" OR "health care utilisation" OR “length of stay” OR hospitalised OR hospitalized)

OR

TI=("healthcare utilization" OR "health care utilization" OR hospitalization OR "healthcare utilisation" OR "health care utilisation" OR “length of stay” OR hospitalised OR hospitalized))))

**PsychInfo**

((((severe mental illness) or (serious mental illness) or schizophrenia or (bipolar disorder)).mp or exp schizophrenia/ or exp bipolar disorder/)

AND

((diabetes OR COPD OR (cardiovascular disease) OR cancer OR (heart disease) OR (atrial fibrillation) or cardiomyopathy OR (liver disease) OR cirrhosis OR stroke OR (chronic obstructive pulmonary) OR hepatitis).mp or exp chronic obstructive pulmonary disease/ or exp diabetes mellitus/ or cardiovascular disorders/ or heart disorders/ or exp neoplasm/ or exp cerebrovascular accident/ or exp liver disorders)

AND

(((admission OR re-admission OR readmission OR attendance OR visit OR admitted) .mp

AND

((emergency department) OR "accident and emergency" OR (secondary care) OR hospital OR inpatient OR outpatient OR planned OR unplanned) .mp)

OR

((healthcare utili#ation) OR (health care utili#ation) OR hospitali#ation OR "length of stay" OR hospitali#ed) .mp))

OR

(((psychosis or psychoses or elixhauser).ti,ab)

AND

((diabetes OR COPD OR (cardiovascular disease) OR cancer OR (heart disease) OR (atrial fibrillation) or cardiomyopathy OR (liver disease) OR cirrhosis OR stroke OR (chronic obstructive pulmonary) OR hepatitis).ti)

AND

(((admission OR re-admission OR readmission OR attendance OR visit OR admitted) .mp

AND

((emergency department) OR "accident and emergency" OR (secondary care) OR hospital OR inpatient OR outpatient OR planned OR unplanned) .mp)

OR

((healthcare utili#ation) OR (health care utili#ation) OR hospitali#ation OR "length of stay" OR hospitali#ed) .mp))

OR

(((psychosis or psychoses).ti)

AND

((diabetes OR COPD OR (cardiovascular disease) OR cancer OR (heart disease) OR (atrial fibrillation) or cardiomyopathy OR (liver disease) OR cirrhosis OR stroke OR (chronic obstructive pulmonary) OR hepatitis).mp)

AND

(((admission OR re-admission OR readmission OR attendance OR visit OR admitted) .mp

AND

((emergency department) OR "accident and emergency" OR (secondary care) OR hospital OR inpatient OR outpatient OR planned OR unplanned) .mp)

OR

((healthcare utili#ation) OR (health care utili#ation) OR hospitali#ation OR "length of stay" OR hospitali#ed) .mp))

**Health management information consortium**

((((severe mental illness) or (serious mental illness) or schizophrenia or (bipolar disorder)).mp or exp "schizophrenia"/ or exp "bipolar disorder"/)

AND

((diabetes OR COPD OR (cardiovascular disease) OR cancer OR (heart disease) OR (atrial fibrillation) OR cardiomyopathy OR (liver disease) OR cirrhosis OR stroke OR (chronic obstructive pulmonary) OR hepatitis).mp or exp chronic obstructive pulmonary disease/ or exp diabetes mellitus/ or cardiovascular diseases/ or heart diseases/ or vascular diseases/ or exp neoplasms/ or exp stroke/ or exp liver diseases)

AND

(((admission OR re-admission OR readmission OR attendance OR visit OR admitted) .mp

AND

((emergency department) OR "accident and emergency" OR (secondary care) OR hospital OR inpatient OR outpatient OR planned OR unplanned) .mp)

OR

((healthcare utili#ation) OR (health care utili#ation) OR hospitali#ation OR "length of stay" OR hospitali#ed) .mp))

OR

(((psychosis or psychoses or elixhauser).ti,ab)

AND

((diabetes OR COPD OR (cardiovascular disease) OR cancer OR (heart disease) OR (atrial fibrillation) OR cardiomyopathy OR (liver disease) OR cirrhosis OR stroke OR (chronic obstructive pulmonary) OR hepatitis).ti)

AND

(((admission OR re-admission OR readmission OR attendance OR visit OR admitted) .mp

AND

((emergency department) OR "accident and emergency" OR (secondary care) OR hospital OR inpatient OR outpatient OR planned OR unplanned) .mp)

OR

((healthcare utili#ation) OR (health care utili#ation) OR hospitali#ation OR "length of stay" OR hospitali#ed) .mp))

OR

(((psychosis or psychoses).ti)

AND

((diabetes OR COPD OR (cardiovascular disease) OR cancer OR (heart disease) OR (atrial fibrillation) OR cardiomyopathy OR (liver disease) OR cirrhosis OR stroke OR (chronic obstructive pulmonary) OR hepatitis).mp)

AND

(((admission OR re-admission OR readmission OR attendance OR visit OR admitted) .mp

AND

((emergency department) OR "accident and emergency" OR (secondary care) OR hospital OR inpatient OR outpatient OR planned OR unplanned) .mp)

OR

((healthcare utili#ation) OR (health care utili#ation) OR hospitali#ation OR "length of stay" OR hospitali#ed) .mp))

**PsychExtra**

((((severe mental illness) or (serious mental illness) or schizophrenia or (bipolar disorder)).mp or exp "schizophrenia"/ or exp "bipolar disorder"/)

AND

((diabetes OR COPD OR (cardiovascular disease) OR cancer OR (heart disease) OR (atrial fibrillation) OR cardiomyopathy OR (liver disease) OR cirrhosis OR stroke OR (chronic obstructive pulmonary) OR hepatitis).mp or exp chronic obstructive pulmonary disease/ or exp diabetes mellitus/ or cardiovascular disorders/ or heart disorders/ or exp neoplasm/ or exp cerebrovascular accident/ or exp liver disorders)

AND

(((admission OR re-admission OR readmission OR attendance OR visit OR admitted) .mp

AND

((emergency department) OR "accident and emergency" OR (secondary care) OR hospital OR inpatient OR outpatient OR planned OR unplanned) .mp)

OR

((healthcare utili#ation) OR (health care utili#ation) OR hospitali#ation OR "length of stay" OR hospitali#ed) .mp))

OR

(((psychosis or psychoses or elixhauser).ti,ab)

AND

((diabetes OR COPD OR (cardiovascular disease) OR cancer OR (heart disease) OR (atrial fibrillation) OR cardiomyopathy OR (liver disease) OR cirrhosis OR stroke OR (chronic obstructive pulmonary) OR hepatitis).ti)

AND

(((admission OR re-admission OR readmission OR attendance OR visit OR admitted) .mp

AND

((emergency department) OR "accident and emergency" OR (secondary care) OR hospital OR inpatient OR outpatient OR planned OR unplanned) .mp)

OR

((healthcare utili#ation) OR (health care utili#ation) OR hospitali#ation OR "length of stay" OR hospitali#ed) .mp))

OR

(((psychosis or psychoses).ti)

AND

((diabetes OR COPD OR (cardiovascular disease) OR cancer OR (heart disease) OR (atrial fibrillation) OR cardiomyopathy OR (liver disease) OR cirrhosis OR stroke OR (chronic obstructive pulmonary) OR hepatitis).mp)

AND

(((admission OR re-admission OR readmission OR attendance OR visit OR admitted) .mp

AND

((emergency department) OR "accident and emergency" OR (secondary care) OR hospital OR inpatient OR outpatient OR planned OR unplanned) .mp)

OR

((healthcare utili#ation) OR (health care utili#ation) OR hospitali#ation OR "length of stay" OR hospitali#ed) .mp))
