# Supplementary material for: The impact of comorbid severe mental illness and common chronic physical health conditions on hospitalisation: A systematic review and meta-analysis
Source: PLoS One. 2022 Aug 18;17(8):e0272498. doi: 10.1371/journal.pone.0272498 (PMC9387848; doi:10.1371/journal.pone.0272498)
Supplement: S1 Table — *Does control for age and is limited to females. (DOCX) [file pone.0272498.s003.docx]

### S2 Table: Study quality and detailed characteristics

| **Authors** | **Year** | **Study region** | **SMI focus** | **Model** | **Matched** | **Controls for** | | | **Denominator source** | **NOS score** | | | |
| --- | --- | --- | --- | --- | --- | --- | --- | --- | --- | --- | --- | --- | --- |
|  |  |  |  |  |  | **Age and sex** | **Physical health comorbidities** | **Prior healthcare use** |  | **Selection** | **Comparability** | **Outcome** | **Total** |
| Helmer et al [31] | 2020 | National | Yes | Logistic regression | No | Yes | Yes | No | Hospital | 2 | 2 | 3 | 7 |
| Tsai et al [33] | 2019 | National | Yes | Cox regression | 1:4 on age, sex, index year, comorbidities | Yes | Yes | No | Hospital, outpatient | 4 | 2 | 2 | 8 |
| Goulesard et al [34] | 2018 | National | Yes | Cox and Logistic regression | No | Yes | Yes | No | Hospital | 2 | 2 | 2 | 6 |
| Edwards et al [35] | 2014 | National | Covariate for different exposure | 2-stage discrete time survival model | No | Yes | Yes | No | Hospital, outpatient, pharmacy | 2 | 2 | 2 | 6 |
| Leung et al [37] | 2011 | One region | Yes | Logistic regression | No | Yes | No | Yes | Hospital, outpatient, pharmacy | 3 | 1 | 3 | 7 |
| Mai et al [38] | 2011 | One region | Yes | Cox regression | 1:2 on age, sex, electoral role | Yes | Yes | No | Hospital, pharmacy, primary care | 4 | 2 | 2 | 8 |
| Attar et al [49] | 2020 | National | Yes | Cox regression | No | Yes | Yes | Yes | Hospital | 4 | 2 | 2 | 8 |
| Chamberlain et al [50] | 2017 | One region | Broad risk factors | Cox regression | No | Yes | Yes | No | Hospital | 3 | 2 | 2 | 7 |
| Basta et al [65] | 2016 | Multiple regions | Broad risk factors | Logistic regression | No | NA* | Yes | No | Hospital | 4 | 2 | 2 | 8 |
| Huckans et al [66] | 2010 | Multiple regions | Yes | None | 1:1 on age, sex, HCV genotype, race | No | No | No | Hospital | 2 | 0 | 3 | 5 |
| Davydow et al [67] | 2016 | National | Yes | None | No | No | No | No | National register | 3 | 0 | 2 | 5 |
| Yan et al [40] | 2019 | Multiple regions | Yes | Logistic regression | No | Yes | Yes | Yes | Hospital, outpatient | 4 | 2 | 3 | 9 |
| Chen et al [41] | 2012 | National | Covariate for different exposure | Logistic regression | No | Yes | Yes | Yes | Hospital | 3 | 2 | 3 | 8 |
| Jorgensen et al [57] | 2017 | National | Yes | Logistic regression | No | Yes | Yes | No | Hospital, outpatient | 4 | 2 | 3 | 9 |
| Ahmedani et al [58] | 2015 | Multiple regions | Yes | None | No | No | No | No | Hospital | 3 | 0 | 3 | 6 |
| Jorgensen et al [62] | 2018 | National | Yes | Logistic regression | No | Yes | Yes | No | Hospital, outpatient | 3 | 2 | 3 | 8 |
| Singh et al [64] | 2016 | National | Yes | Multi-level logistic regression | No | Yes | No | No | Hospital | 2 | 1 | 3 | 6 |
| Guerrero Fernandez de Alba et al [42] | 2020 | One region | Yes | Logistic regression | No | Yes | Yes | No | Hospital, pharmacy, primary care | 2 | 2 | 2 | 6 |
| Chwastiak et al [43] | 2014 | One region | Yes | Cox and Logistic regression | No | Yes | Yes | Yes | Hospital | 3 | 2 | 2 | 7 |
| Becker et al [44] | 2011 | One region | Yes | Cox regression | 1:3 on age, sex, region, socioeconomic status | Yes | Yes | Yes | Hospital, physician visits | 4 | 2 | 2 | 8 |
| Lu et al [60] | 2017 | Single site | Yes | Cox regression | No | Yes | Yes | No | Hospital | 2 | 2 | 3 | 7 |
| Guo et al [68] | 2008 | Multiple regions | Yes | Poisson regression | No | Yes | Yes | No | Hospital, pharmacy, outpatient, primary care | 2 | 2 | 2 | 6 |
| Kurdyak et al [46] | 2017 | One region | Yes | Logistic regression | No | Yes | Yes | No | Hospital, physician visits | 2 | 2 | 2 | 6 |
| Shim et al [47] | 2014 | Multiple regions | Yes | None | No | No | No | No | Hospital, outpatient | 2 | 0 | 2 | 4 |
| Egglefield et al [30] | 2020 | One region | Yes | None | No | No | No | No | Hospital, outpatient, pharmacy | 2 | 0 | 2 | 4 |
| Stockbridge et al [32] | 2019 | National | Yes | Negative binomial-logit hurdle regression | No | Yes | Yes | No | Hospital, pharmacy | 2 | 2 | 3 | 7 |
| Druss et al [36] | 2012 | National | Yes | Generalised linear mixed model | No | Yes | Yes | No | Hospital, outpatient | 2 | 2 | 3 | 7 |
| Cramer et al [39] | 2010 | National | Broad risk factors | Logistic regression | No | No | Yes | No | Hospital | 1 | 1 | 3 | 5 |
| Sayers et al [51] | 2007 | National | Yes | Generalised linear modelling | No | Yes | Yes | No | Hospital | 2 | 2 | 3 | 7 |
| Shah et al [52] | 2018 | Multiple regions | Broad risk factors | Multi-level logistic regression | No | Yes | Yes | No | Hospital | 3 | 2 | 3 | 8 |
| Pham et al [53] | 2019 | Multiple regions | Broad risk factors | Multi-level logistic regression | No | Yes | Yes | No | Hospital | 2 | 2 | 3 | 7 |
| Chamberlain et al [54] | 2018 | Multiple regions | Broad risk factors | Logistic regression | No | Yes | Yes | No | Hospital | 3 | 2 | 3 | 8 |
| Shah et al [55] | 2018 | Multiple regions | Broad risk factors | Multi-level logistic regression | No | Yes | Yes | No | Hospital | 3 | 2 | 3 | 8 |
| Shah et al [56] | 2018 | Multiple regions | Broad risk factors | Multi-level logistic regression | No | Yes | Yes | No | Hospital | 3 | 2 | 3 | 8 |
| Coffey et al [59] | 2012 | Multiple regions | Broad risk factors | Multi-level logistic regression | No | Yes | Yes | No | Hospital | 2 | 2 | 3 | 7 |
| Buhr et al [61] | 2019 | National | Broad risk factors | None | No | No | No | No | Hospital | 3 | 0 | 2 | 5 |
| Lau et al [63] | 2017 | Multiple regions | Broad risk factors | Logistic regression | No | Yes | Yes | No | Hospital | 3 | 2 | 3 | 8 |
| Krein et al [45] | 2006 | National | Yes | None | 1:1 on age | No | No | No | Hospital, outpatient, pharmacy | 2 | 0 | 2 | 4 |
| Sullivan et al [48] | 2006 | Single site | Yes | Generalised estimating equation | No | Yes | No | No | Hospital | 2 | 1 | 3 | 6 |
| Wang et al [79] | 2021 | National | Yes | Generalised linear model | Up to 1: 4 on age, sex and primary care practice | Yes | Yes | Yes | Primary care | 4 | 2 | 3 | 9 |
| Kashyap et al [69] | 2021 | National | Yes | Logistic regression | No | Yes | Yes | No | Hospital | 3 | 2 | 3 | 8 |
| Kallio et al [70] | 2022 | Multi-centre | Yes | None | Propensity score, 1:20 | No | No | No | Hospital | 3 | 0 | 3 | 6 |
| Fleetwood et al [72] | 2021 | National | Yes | Competing risk survival analysis | No | Yes | No | No | Hospital | 4 | 1 | 3 | 8 |
| Ghani et al [73] | 2021 | One region | Yes | Logistic regression | No | Yes | No | Yes | Hospital | 2 | 1 | 3 | 6 |
| Fleetwood et al [71] | 2021 | National | Yes | Competing risk survival analysis | No | Yes | No | No | Hospital | 4 | 1 | 3 | 8 |
| Huang et al [74] | 2021 | National | Yes | None | Propensity score, 1:10 | No | No | No | Primary care and hospital | 4 | 0 | 3 | 7 |
| Ratcliff et al [75] | 2021 | National | Yes | None | No | No | No | No | Hospital | 3 | 0 | 3 | 6 |
| Paredes et al [76] | 2020 | National | Yes | Logistic regression | No | Yes | Yes | No | Hospital | 2 | 2 | 3 | 7 |
| Sreenivasan et a [77]l | 2022 | National | Yes | Cox regression | No | Yes | Yes | No | Hospital | 3 | 2 | 3 | 8 |
| Andres et al [78] | 2012 | One region | Yes | None | No | No | No | No | Hospital | 3 | 0 | 3 | 6 |

*Does control for age, and is limited to females.
