# Supplementary material for: The impact of comorbid severe mental illness and common chronic physical health conditions on hospitalisation: A systematic review and meta-analysis
Source: PLoS One. 2022 Aug 18;17(8):e0272498. doi: 10.1371/journal.pone.0272498 (PMC9387848; doi:10.1371/journal.pone.0272498)
Supplement: S2 Table — *One point. a: Analysis presenting odds ratios; b: analysis presenting hazard ratios c: Analysis of 30-day readmissions; d: analysis of long-term readmissions. (DOCX) [file pone.0272498.s004.docx]

### S3 Table: Components of the Newcastle-Ottawa Score

| **Authors** | **Year** | **Selection** | | | | **Comparability** | | **Outcome** | | | **Total number of stars** |
| --- | --- | --- | --- | --- | --- | --- | --- | --- | --- | --- | --- |
|  |  | **Exposed cohort representative** | **Selection of non-exposed** | **Ascertainment of exposure** | **Outcome not present at start** | **Controls for age and sex** | **Controls for physical health comorbidities** | **Assessment of outcome** | **Length of follow up** | **Lost to follow up** |  |
| **Cohort studies** | | | | | | | | | | | |
| Helmer et al[31] | 2020 | Select group | Same setting* | Secure record* | No | Yes* | Yes* | Record linkage* | Adequate* | Complete follow up* | 7 |
| Tsai et al[33] | 2019 | Truly* | Same setting* | Secure record* | Yes* | Yes* | Yes* | Record linkage* | Adequate* | May introduce bias | 8 |
| Goulesard et al[34] | 2018 | Select group | Same setting* | Secure record* | No | Yes* | Yes* | Record linkage* | Adequate* | May introduce bias | 6 |
| Edwards et al[35] | 2014 | Select group | Same setting* | Secure record* | No | Yes* | Yes* | Record linkage* | Adequate* | No description | 6 |
| Leung et al[37] | 2011 | Select group | Same setting* | Secure record* | Yes* | Yes* | No | Record linkage* | Adequate* | Satisfactory* | 7 |
| Mai et al[38] | 2011 | Somewhat* | Same setting* | Secure record* | Yes* | Yes* | Yes* | Record linkage* | Adequate* | No description | 8 |
| Attar et al[49] | 2020 | Truly* | Same setting* | Secure record* | Yes* | Yes* | Yes* | Record linkage* | Adequate* | May introduce bias | 8 |
| Chamberlain et al[50] | 2017 | Select group | Same setting* | Secure record* | Yes* | Yes* | Yes* | Record linkage* | Adequate* | No description | 7 |
| Basta et al[65] | 2016 | Somewhat* | Same setting* | Secure record* | Yes* | NA* | Yes* | Record linkage* | Adequate* | May introduce bias | 8 |
| Huckans et al[66] | 2010 | Select group | Same setting* | Secure record* | No | No | No | Record linkage* | Adequate* | Satisfactory* | 5 |
| Davydow et al[67] | 2016 | Truly* | Same setting* | Secure record* | No | No | No | Record linkage* | Adequate* | No description | 5 |
| Yan et al[40] | 2019 | Somewhat* | Same setting* | Secure record* | Yes* | Yes* | Yes* | Record linkage* | Adequate* | Complete follow up* | 9 |
| Chen et al[41] | 2012 | Select group | Same setting* | Secure record* | Yes* | Yes* | Yes* | Record linkage* | Adequate* | Complete follow up* | 8 |
| Jorgensen et al[57] | 2017 | Truly* | Same setting* | Secure record* | Yes* | Yes* | Yes* | Record linkage* | Adequate* | Satisfactory* | 9 |
| Ahmedani et al[58] | 2015 | Select group | Same setting* | Secure record* | Yes* | No | No | Record linkage* | Adequate* | Satisfactory* | 6 |
| Jorgensen et al[62] | 2018 | Truly* | Same setting* | Secure record* | No | Yes* | Yes* | Record linkage* | Adequate* | Satisfactory* | 8 |
| Singh et al[64] | 2016 | Select group | Same setting* | Secure record* | No | Yes* | No | Record linkage* | Adequate* | Satisfactory* | 6 |
| Guerrero Fernandez de Alba et al[42] | 2020 | Select group | Same setting* | Secure record* | No | Yes* | Yes* | Record linkage* | Adequate* | No description | 6 |
| Chwastiak et al[43]^a^ | 2014 | Select group | Same setting* | Secure record* | Yes* | Yes* | Yes* | Record linkage* | Adequate* | Satisfactory* | 8 |
| Chwastiak et al[43]^b^ | 2014 | Select group | Same setting* | Secure record* | Yes* | Yes* | Yes* | Record linkage* | Adequate* | No description | 7 |
| Becker et al[44] | 2011 | Somewhat* | Same setting* | Secure record* | Yes* | Yes* | Yes* | Record linkage* | Adequate* | May introduce bias | 8 |
| Lu et al[60]^c^ | 2017 | Select group | Same setting* | Secure record* | No | Yes* | Yes* | Record linkage* | Adequate* | Satisfactory* | 7 |
| Lu et al[60]^d^ | 2017 | Select group | Same setting* | Secure record* | No | Yes* | Yes* | Record linkage* | Adequate* | May introduce bias | 6 |
| Guo et al[68] | 2008 | Select group | Same setting* | Secure record* | No | Yes* | Yes* | Record linkage* | Adequate* | No description | 6 |
| Kurdyak et al[46] | 2017 | Select group | Same setting* | Secure record* | No | Yes* | Yes* | Record linkage* | Adequate* | No description | 6 |
| Shim et al[47] | 2014 | Select group | Same setting* | Secure record* | No | No | No | Record linkage* | Adequate* | No description | 4 |
| Wang et al [79] |  | Truly* | Same setting* | Secure record* | a | Yes* | Yes* | Record linkage* | Adequate* | Satisfactory* | 9 |
| Kashyap et al [69] |  | Select group | Same setting* | Secure record* | a | Yes* | Yes* | Record linkage* | Adequate* | Satisfactory* | 8 |
| Kallio et al [70] |  | Select group | Same setting* | Secure record* | a | No | No | Record linkage* | Adequate* | Satisfactory* | 6 |
| Fleetwood et al [72] |  | Truly* | Same setting* | Secure record* | Yes* | Yes* | No | Record linkage* | Adequate* | Satisfactory* | 8 |
| Ghani et al [73] |  | Select group | Same setting* | Secure record* | b | Yes* | No | Record linkage* | Adequate* | Satisfactory* | 6 |
| Fleetwood et al [71] |  | Truly* | Same setting* | Secure record* | Yes* | Yes* | No | Record linkage* | Adequate* | Satisfactory* | 8 |
| Huang et al [74] |  | Truly* | Same setting* | Secure record* | Yes* | No | No | Record linkage* | Adequate* | Satisfactory* | 7 |
| Ratcliff et al [75] |  | Select group | Same setting* | Secure record* | Yes* | No | No | Record linkage* | Adequate* | Satisfactory* | 6 |
| Paredes et al [76] |  | Select group | Same setting* | Secure record* | b | Yes* | Yes* | Record linkage* | Adequate* | Satisfactory* | 7 |
| Sreenivasan et al [77] |  | Truly* | Same setting* | Secure record* | b | Yes* | Yes* | Record linkage* | Adequate* | Satisfactory* | 8 |
| **Cross-sectional studies** | | | | | | | | | | | |
|  |  | **Selection** | | | | **Comparability** | | **Outcome** | | |  |
|  |  | **Sample representative** | **Sample size** | **Ascertainment of exposure** | **Non-response rate** | **Controls for age and sex** | **Controls for physical health comorbidities** | **Assessment of outcome** | **Statistical test** | **Time period** | **Total** |
| Egglefield et al[30] | 2020 | Select group | Justified* | Secure record* | No description | No | No | Record linkage* | Not appropriate | Appropriate* | 4 |
| Stockbridge et al[32] | 2019 | Select group | Justified* | Secure record* | No description | Yes* | Yes* | Record linkage* | Appropriate* | Appropriate* | 7 |
| Druss et al[36] | 2012 | Select group | Justified* | Secure record* | No description | Yes* | Yes* | Record linkage* | Appropriate* | Appropriate* | 7 |
| Cramer et al[39] | 2010 | Select group | Unjustified | Secure record* | No description | No | Yes* | Record linkage* | Appropriate* | Appropriate* | 5 |
| Sayers et al[51] | 2007 | Select group | Justified* | Secure record* | No description | Yes* | Yes* | Record linkage* | Appropriate* | Appropriate* | 7 |
| Shah et al[52] | 2018 | Somewhat* | Justified* | Secure record* | No description | Yes* | Yes* | Record linkage* | Appropriate* | Appropriate* | 8 |
| Pham et al[53] | 2019 | Select group | Justified* | Secure record* | No description | Yes* | Yes* | Record linkage* | Appropriate* | Appropriate* | 7 |
| Chamberlain et al[54] | 2018 | Somewhat* | Justified* | Secure record* | No description | Yes* | Yes* | Record linkage* | Appropriate* | Appropriate* | 8 |
| Shah et al[55] | 2018 | Somewhat* | Justified* | Secure record* | No description | Yes* | Yes* | Record linkage* | Appropriate* | Appropriate* | 8 |
| Shah et al[56] | 2018 | Somewhat* | Justified* | Secure record* | No description | Yes* | Yes* | Record linkage* | Appropriate* | Appropriate* | 8 |
| Coffey et al[59] | 2012 | Somewhat* | Unjustified | Secure record* | No description | Yes* | Yes* | Record linkage* | Appropriate* | Appropriate* | 7 |
| Buhr et al[61] | 2019 | Somewhat* | Justified* | Secure record* | No description | No | No | Record linkage* | Not appropriate | Appropriate* | 5 |
| Lau et al[63] | 2017 | Somewhat* | Justified* | Secure record* | No description | Yes* | Yes* | Record linkage* | Appropriate* | Appropriate* | 8 |
| Krein et al[45] | 2006 | Select group | Justified* | Secure record* | No description | No | No | Record linkage* | Not appropriate | Appropriate* | 4 |
| Sullivan et al[48] | 2006 | Select group | Justified* | Secure record* | No description | Yes* | No | Record linkage* | Appropriate* | Appropriate* | 6 |
| Andres et al [78] | 2012 | Somewhat* | Justified* | Secure record* | No description | No | No | Record linkage* | Appropriate* | Appropriate* | 6 |

*One point

a: Analysis presenting odds ratios; b: analysis presenting hazard ratios c: Analysis of 30-day readmissions; d: analysis of long term readmissions.
