# Supplementary material for: The impact of comorbid severe mental illness and common chronic physical health conditions on hospitalisation: A systematic review and meta-analysis
Source: PLoS One. 2022 Aug 18;17(8):e0272498. doi: 10.1371/journal.pone.0272498 (PMC9387848; doi:10.1371/journal.pone.0272498)

### S4 Figure: Funnel plots for all individual analyses

A: Studies which present odds ratios


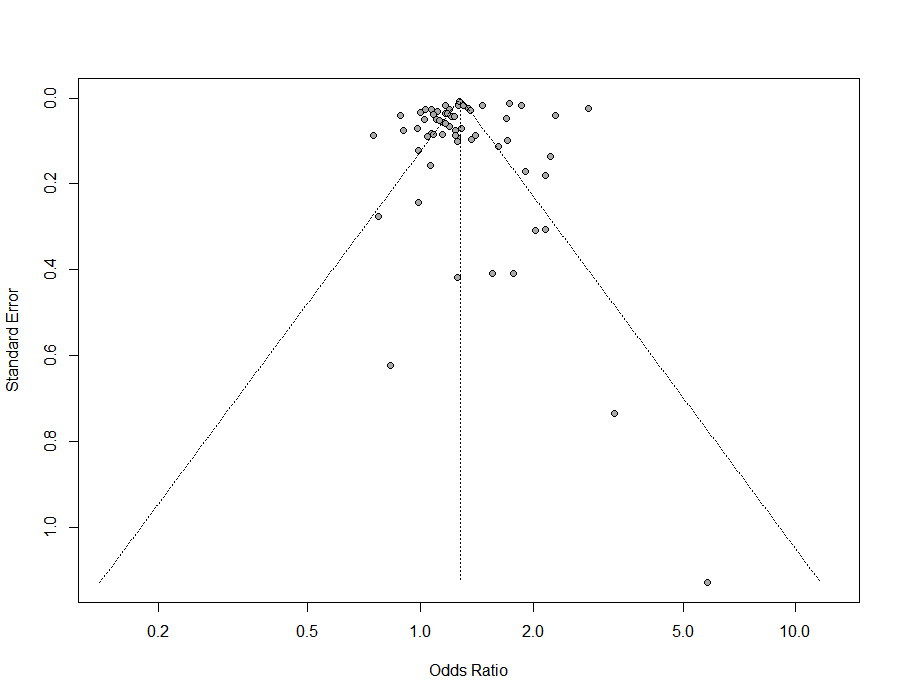


B: Studies which present hazard ratios


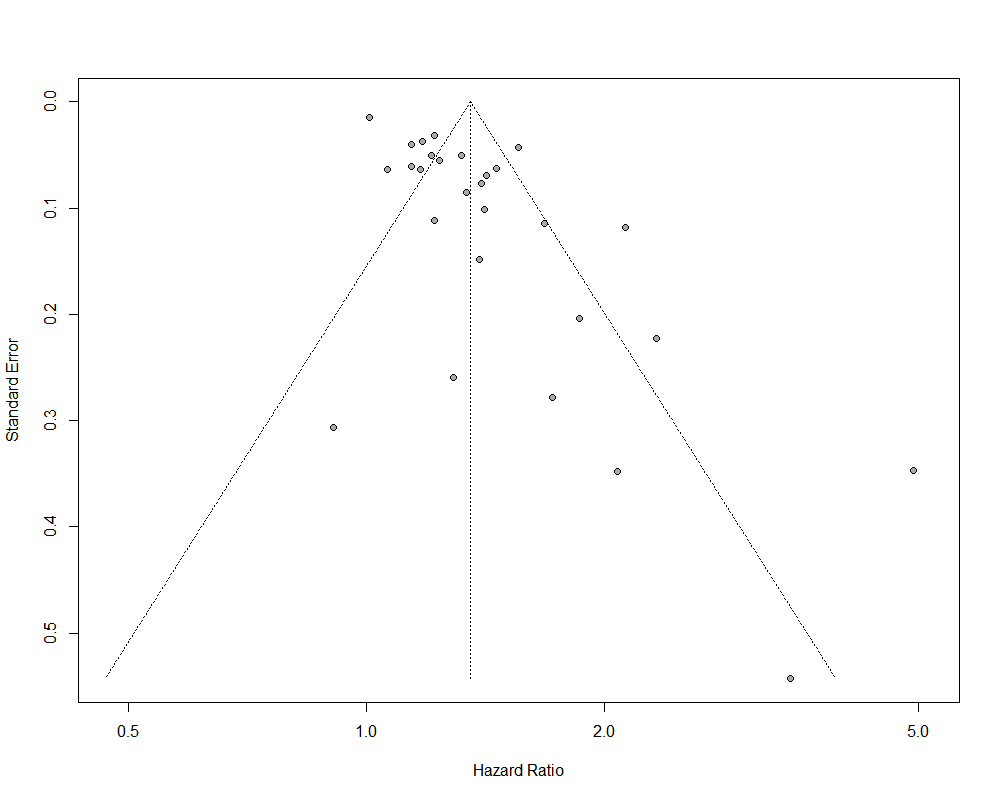


C: Studies which present risk ratios


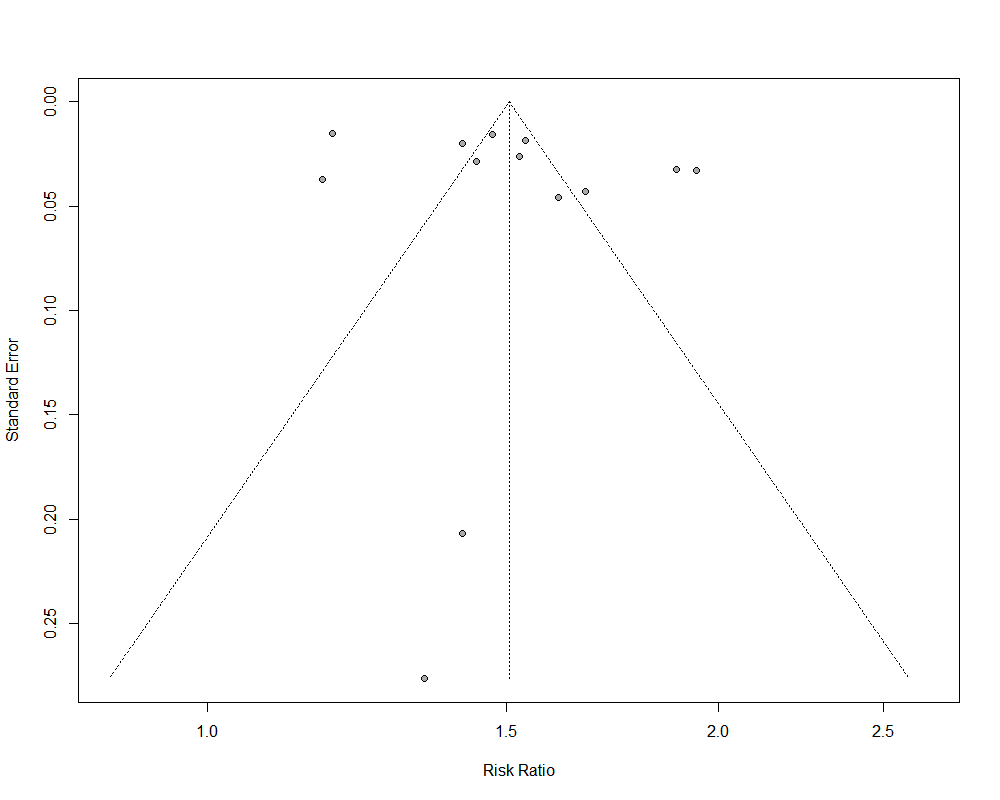

Supplement: S1 Fig — (DOCX) [file pone.0272498.s005.docx]
