# Supplementary material for: The impact of comorbid severe mental illness and common chronic physical health conditions on hospitalisation: A systematic review and meta-analysis
Source: PLoS One. 2022 Aug 18;17(8):e0272498. doi: 10.1371/journal.pone.0272498 (PMC9387848; doi:10.1371/journal.pone.0272498)

### S8 Figure: Funnel plot for studies presenting adjusted odds ratios of hospital utilisation in COPD patients with SMI compared to without SMI


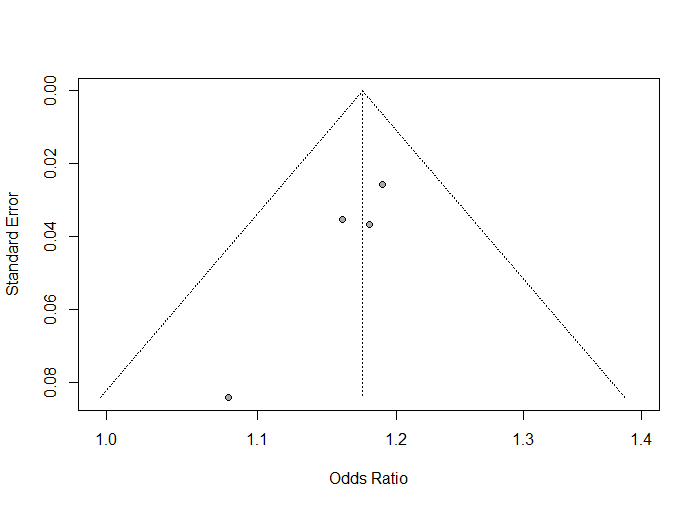

Supplement: S6 Fig — (DOCX) [file pone.0272498.s010.docx]
