# Supplementary material for: The impact of comorbid severe mental illness and common chronic physical health conditions on hospitalisation: A systematic review and meta-analysis
Source: PLoS One. 2022 Aug 18;17(8):e0272498. doi: 10.1371/journal.pone.0272498 (PMC9387848; doi:10.1371/journal.pone.0272498)
Supplement: Attachment — Submitted filename: Response to reviewers.docx [file pone.0272498.s011.docx]

**Editor comments to the author**

**Please ensure that your manuscript meets PLOS ONE's style requirements**

We have reviewed the style requirements and made changes where necessary.

**Please remove any funding-related text from the manuscript and let us know how you would like to update your Funding Statement.**

This has now been removed. Could we please have the following text in our funding statement:

“This study was supported by Public Health England (PhD2019/002 - NL), the Wellcome Trust (211085/Z/18/Z - JFH), the Medical Research Council (MC\PC\17216 - DPJO), University College London Hospitals NIHR Biomedical Research Centre (NL, DPJO, JFH) and the NIHR ARC North Thames Academy (DPJO, JFH). This report is independent research supported by the National Institute for Health Research ARC North Thames. The funders had no role in study design, data collection and analysis, decision to publish, or preparation of the manuscript. The views expressed in this publication are those of the author(s) and not necessarily those of the National Institute for Health Research, Public Health England, or the Department of Health and Social Care.”

**Please note that in order to use the direct billing option the corresponding author must be affiliated with the chosen institute. Please either amend your manuscript to change the affiliation or corresponding author, or email us at** [**plosone@plos.org**](mailto:plosone@plos.org) **with a request to remove this option.**

We have emailed [plosone@plos.org](mailto:plosone@plos.org) about this. We may have chosen the wrong option on the submission form. We believe that UCL have a transformative agreement with the PLOS journals.

**Reviewer comments to the author**

Reviewer #1: The present Systematic Review and Meta-analysis aimed at identifying the hospital utilization's rates in people affected by SMI with a comorbid physical health condition. The topic is interesting and the authors made a huge effort in summarizing the available evidence, choosing multiple outcomes and health conditions.
I have a few comments that, in my opinion, may help to improve or clarify the manuscript:

**- The search was conducted until March 2020. Since 2 years have passed, I think that the authors should update their original search to detect the most current studies on the topic.**

Thank you for this comment. The submitted manuscript included searches run in March and December 2020. We agree that it would be beneficial to re-run the searches. Searches were therefore re-run in March 2022. We identified 11 new studies. Two new studies have been added to the existing meta-analysis of heart disease and one to the diabetes aOR meta-analysis. Three new studies in patients with heart disease presented aHR and so it was possible to do a meta-analysis of aHR for heart disease. These changes are in track changes throughout the text. While re running the searches identified new papers, it did not change the conclusions of the meta analyses or the review as a whole.

**Additionally, I suggest to put the complete data (dd/mm/yyyy) in the main text to allow an easier reproducibility;**

Dates of all searches are now included in the text in lines 72 and 75:

“We searched the following sources on 24 March 2020 for publications or grey literature within the remit of the study without date restrictions: PubMED, EmBase, Web of Science, PsychInfo, PsychExtra, Health Management Information Centre. Searches for new publications were performed on 17 December 2020 and 17 March 2022.”

**- Which definition of SMI did the authors adopt? I think that they should specify how they define SMI. For example, they excluded the MDD from their SMI definition (line 89), while it is included as SMI in other studies;**

We focused on studies including patients with a diagnosis of schizophrenia, bipolar disorder or other non-organic psychoses, in line with the Quality Outcomes Framework in the UK. This is in line with the majority of studies we identified. We excluded the small number of studies which included MDD in their definition. This is now clarified in the text on lines 90-94:

“We defined SMI as patients with a diagnosis of either schizophrenia, bipolar disorder or other non-organic long-term psychotic disorders, in line with the Quality Outcomes Framework used by the NHS in England[25]. We therefore excluded studies that included major depression in their definition of SMI, without stratifying results by mental health condition.”

**- Did you include only those studies adopting DSM/ICD criteria to make a diagnosis of SMI? If not, why?**

We did not limit the selection based on those studies adopting DSM/ICD criteria to make a diagnosis of SMI. Most studies used data retrieved from medical notes. While some studies detailed how the diagnosis was made, for many it was either unknown or unclear.

**- Line 117, the authors used a score of 6 as a cut-off to divide low and high-risk of bias studies. I think that the authors should motivate their decision as it could seem arbitrary (i.e., citing other studies adopting the same method);**

On reviewing the rationale for this, it is evident that studies use a variety of cut-offs to determine study inclusion and/or study quality. As such we have removed this statement from the methods and included all studies providing relevant adjusted effect measures in the meta-analysis. This change only results in the addition of one study to the meta analysis of ORs in patients with diabetes.

**- The Cohen's Kappa is 0.57 that is quite low compared to other reviews. I think that the authors should double-check this passage and consider to add it as a limitation;**

We have double checked the calculation and it is correct. We have added the following to the limitations (line 380-385):

“In addition, while our search strategy was thorough, and overall agreement between reviewers was high (91%), the interrater reliability of screened abstracts as measured by the Kappa statistic was moderate (0.57). This is in part due to the large number of studies screened and the rarity of relevant studies[92], but also the complexity of multiple exposures and outcomes. All disagreements were discussed thoroughly to ensure the accuracy of study inclusion.”

**- Table 1, I think it could be useful to add a column in which reporting the study design;**

Study design has now been added to Table 1.

**- Line 308, could you please rephrase this sentence? It appears quite unclear to me**

We have reworded this (now on line x) to read: “A strength of focusing on studies in populations with underlying physical LTC, is that it provides further evidence that the higher emergency hospitalisation in people with SMI is not due to higher prevalence of that LTC in the SMI population.”

**Reviewer #2: The authors appropriately performed statistical analysis. The results are exhaustively presented and adequately discussed in the light of the most recent literature evidence. Compared to the relevant meta-analysis performed by Ronaldson A. et al. (2020), the present MA has the advantage of assessing the outcomes separately for the five medical comorbidities examined, thus adding a relevant contribution to the field.**

We thank the reviewer for their comments.
